# Persistent Regulation of Tumor Hypoxia Microenvironment via a Bioinspired Pt‐Based Oxygen Nanogenerator for Multimodal Imaging‐Guided Synergistic Phototherapy

**DOI:** 10.1002/advs.201903341

**Published:** 2020-07-29

**Authors:** Qing You, Kaiyue Zhang, Jingyi Liu, Changliang Liu, Huayi Wang, Mengting Wang, Siyuan Ye, Houqian Gao, Letian Lv, Chen Wang, Ling Zhu, Yanlian Yang

**Affiliations:** ^1^ CAS Key Laboratory of Standardization and Measurement for Nanotechnology CAS Key Laboratory for Biomedical Effects of Nanomaterials and Nanosafety CAS Center for Excellence in Nanoscience National Center for Nanoscience and Technology Beijing 100190 China; ^2^ University of Chinese Academy of Sciences Beijing 100049 P. R. China; ^3^ Sino‐Danish College University of Chinese Academy of Sciences Beijing 100049 P. R. China; ^4^ Department of Chemistry Tinghua University Beijing 100084 P. R. China

**Keywords:** cancer theranostics, hypoxia alleviation, multimodal imaging, O_2_ self‐enriched photodynamic therapy, photothermal therapy

## Abstract

Multifunctional nanoplatforms for imaging‐guided synergistic antitumor treatment are highly desirable in biomedical applications. However, anticancer treatment is largely affected by the pre‐existing hypoxic tumor microenvironment (TME), which not only causes the resistance of the tumors to photodynamic therapy (PDT), but also promotes tumorigenesis and tumor progression. Here, a continuous O_2_ self‐enriched nanoplatform is constructed for multimodal imaging‐guided synergistic phototherapy based on octahedral gold nanoshells (GNSs), which are constructed by a more facile and straightforward one‐step method using platinum (Pt) nanozyme‐decorated metal–organic frameworks (MOF) as the inner template. The Pt‐decorated MOF@GNSs (PtMGs) are further functionalized with human serum albumin‐chelated gadolinium (HSA‐Gd, HGd) and loaded with indocyanine green (ICG) (ICG‐PtMGs@HGd) to achieve a synergistic PDT/PTT effect and fluorescence (FL)/multispectral optoacoustic tomography (MSOT)/X‐ray computed tomography (CT)/magnetic resonance (MR) imaging. The Pt‐decorated nanoplatform endows remarkable catalase‐like behavior and facilitates the continuous decomposition of the endogenous H_2_O_2_ into O_2_ to enhance the PDT effect under hypoxic TME. HSA modification enhances the biocompatibility and tumor‐targeting ability of the nanocomposites. This TME‐responsive and O_2_ self‐supplement nanoparticle holds great potential as a multifunctional theranostic nanoplatform for the multimodal imaging‐guided synergistic phototherapy of solid tumors.

## Graphical abstract

In this research, a tumor environment (TEM)‐responsive and continuous O_2_ self‐enriched drug delivery platform based on octahedral gold nanoshells (GNSs) using Pt‐decorated metal–organic frameworks (MOF) as inner template is constructed. The obtained nanostructures are further functionalized with human serum albumin‐gadolinium hybrid (HSA‐Gd, HGd) and loaded with indocyanine green (ICG) (ICG‐PtMGs@HGd) to achieve a multimodal imaging guided enhanced photodynamic therapy/photothermal therapy PDT/PTT effect.

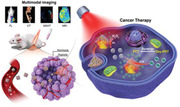

## 1 Introduction

Nanotheranostic platforms that combine the multiple antitumor therapies under the guidance of multimodal imaging have become the trend in cancer therapy due to their enhanced therapeutic effect, improved tumor specificity, reduced drug resistance, and the convenience for the precise tumor lesion detection and treatment monitoring.^[^ ^1^ ^]^ With the rapid development of nanomedicine, a variety of treatment modalities have been investigated, among which phototherapy has attracted wide attention due to its minimal invasiveness, high tumor specificity, excellent spatial/temporal controllability, and less toxicity.^[^ ^2^ ^]^ Photodynamic therapy (PDT) and photothermal therapy (PTT) are the two main phototherapies. PDT utilizes the photosensitizers (PS) to generate reactive oxygen species (ROS) under light irradiation to kill the tumor cells while PTT enables the tumor death via the local hyperthermia induced by the photothermal agents under light irradiation.^[^ ^3^ ^]^ Multifunctional nanoparticles enabling the combinatorial effects of PDT and PTT, and the simultaneous in situ imaging such as the optical imaging, photoacoustic tomography (PA), X‐ray computed tomography (CT), and magnetic resonance imaging (MRI) have been constructed.^[^ ^4^ ^]^ However, the anticancer therapy of such high‐performance nanoplatforms remains intractable challenging due to the complex tumor microenvironment (TME) and the induced therapeutic resistances.

Hypoxic microenvironment is one of the typical characteristics of the solid tumors owing to the imbalance between the high oxygen consumption caused by the fast proliferation of the tumor cells and the inefficient supply of oxygen caused by the malformation of the tumor vascular systems.^[^ ^5^ ^]^ Hypoxia not only caused the resistance of the tumors to PDT that requires high concentration of oxygen to generate the cytotoxic ROS, but also promoted the tumorigenesis and tumor progression as a result of DNA methylation and the high expression of hypoxia‐inducible factors (HIFs).^[^ ^6^ ^]^ Meanwhile, the unexpected tumor metabolites such as hydrogen peroxide (H_2_O_2_) are also generated under the hypoxia condition, which promotes the invasiveness and metastasis of cancer.^[^ ^7^ ^]^ Many efforts have been devoted to alleviate the hypoxia and to improve the PDT effect, including the hyperbaric oxygen therapy,^[^ ^8^ ^]^ the oxygen carriers (e.g., hemoglobin and perfluorocarbon)^[^ ^9^ ^]^ and the catalyze‐like oxygen generators (e.g., manganese dioxide (MnO_2_) and copper oxide (CuO)).^[^ ^10^ ^]^ Among them, utilizing the nanozyme as catalysts to generate oxygen in situ, has attracted increasing interests due to its resistance to the stringent conditions, the tunable enzymatic activity, and the low production cost.^[^ ^11^ ^]^ Many noble metal nanomaterials have been used as catalytic nanozymes for oxygen generation to enhance ROS production in TME,^[^ ^12^ ^]^ among which platinum (Pt)^[^ ^13^ ^]^ and palladium (Pd)^[^ ^14^ ^]^ nanoparticles exhibit durable catalytic and antioxidant properties and serves as a desire catalyst to continuously decompose H_2_O_2_ into O_2_ without rapid self‐consumption as was common for the other catalysts such as MnO_2_.^[^ ^15^ ^]^

**Scheme 1:**
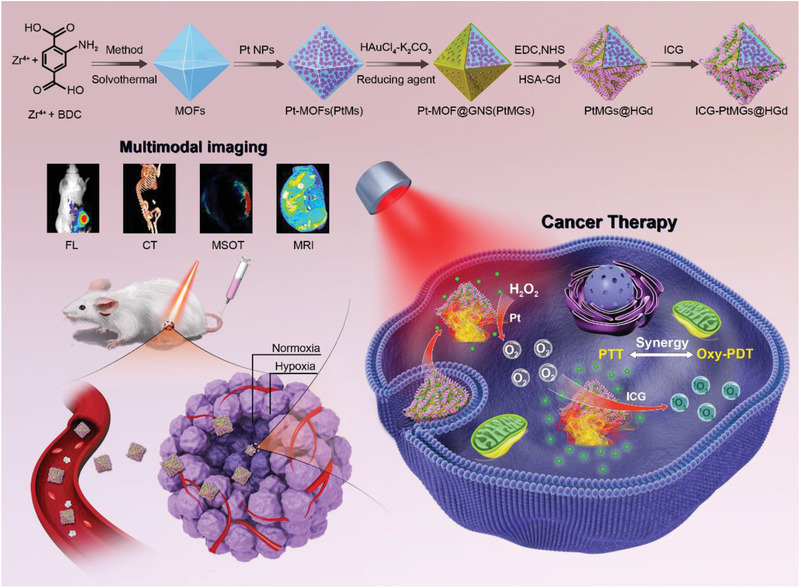
Schematic illustration of the ICG‐PtMGs@HGd nanoplatforms as H_2_O_2_‐driven oxygenator for FL/MOST/CT/MRI multimodal imaging guided enhanced PDT and PTT synergistic therapy in a solid tumor.

Here, we develop a hypoxia‐resistant and persistent O_2_ self‐supplement nanoplatform to achieve an improved synergistic phototherapy with the guidance of FL/MSOT/CT/MR quadruple‐modal imaging based on Pt‐decorated gold nanoparticles using metal–organic frameworks (MOFs) as the inner template (**Scheme** **1**). MOFs, the crystalline porous hybrids built by bridging the metal ions/clusters with the organic ligands, have shown great potential in drug delivery due to their well‐defined pore aperture, intrinsic biodegradability, facile and designable synthesis, and tunable sizes/shapes.^[^ ^16^ ^]^ The stable crystalline porous structure of MOFs enables the loading of large amount of Pt nanozymes. On the other hand, porous gold nanoshells (GNSs) have been widely applied in PTT owing to their surface plasmon resonance (SPR) ability and the porous structures for the synergistic therapy with other modalities.^[^ ^1a^ ^]^ The GNSs also possess ideal X‐ray attenuation coefficient, holding great potential for CT imaging.^[^ ^17^ ^]^ The conventional method for preparing GNSs is to deposit the gold materials onto the surfaces of the suitable templates, such as the mesoporous silica^[^ ^18^ ^]^ and the metal nanoparticles (e.g., cobalt^[^ ^19^ ^]^ and silver^[^ ^20^ ^]^ nanoparticles), which requires the complicated synthetic procedure and the strict reaction conditions. In this regard, the tailorable synthesis and the tunable pore aperture of MOF make it a promising template to construct GNSs. In our design, the porous GNSs were constructed by a rapid and straightforward one‐step method^[^ ^21^ ^]^ in which the gold was grafted on the template of Pt‐loaded MOFs (Pt‐MOF@GNSs, PtMGs). The obtained PtMGs were further decorated with human serum albumin (HSA)‐gadolinium (Gd) hybrids (HSA‐Gd, HGd) (PtMG@HGd)^[^ ^22^ ^]^ to enable MR imaging utilizing the high longitudinal proton relaxivity of Gd.^[^ ^23^ ^]^ Meanwhile, the coating of endogenous HSA that is approved by the U.S. Food and Drug Administration (FDA) for intravenous administration increases the biocompatibility, stability, and tumor targeting effect (passive or active) of the nanoplatforms.^[^ ^24^ ^]^ Finally, indocyanine green (ICG), a commercially NIR organic dye that has been approved by FDA is loaded into the nanoplatform, endowing the nanocarriers synergistic PDT/PTT effect as well as fluorescence (FL)/multispectral optoacoustic tomography (MSOT) imaging.^[^ ^25^ ^]^

The as prepared ICG‐PtMG@HGd nanocomposites enable persistent regulation of the hypoxia microenvironment in the tumor by the Pt‐induced self‐supply of oxygen, and exhibit improved effects in the oncolysis and metastasis inhibition of the tumor by the synergistic phototherapy. The simultaneously obtained FL/MSOT/CT/MR quadruple‐modal imaging provides more accurate information of the tumor from the optical, electronic, and magnetic perspectives. This versatile nanotheranostic platform shows its advantages in the treatment and in situ imaging of the tumor, provides insights into design of the multifunctional nanoparticles for TME‐responsive cancer therapy.

## 2 Results

### 2.1 Synthesis and Characterization of ICG‐PtMGs@HGd

**Figure 1:**
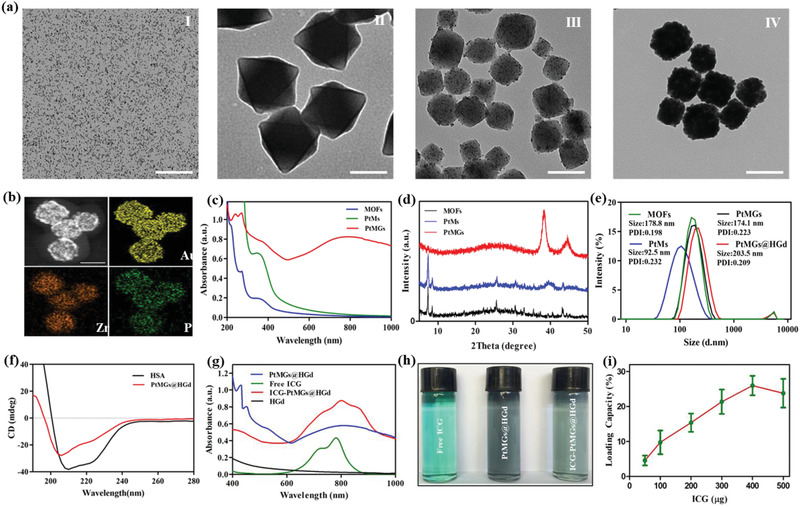
Synthesis and characterization of ICG‐PtMGs@HGd. a) TEM images of the different nanoparticles during the process of the PtMGs: Pt nanoparticles (I), MOFs (II), PtMs (III), and PtMGs (IV), respectively. Scale bar, 100 nm. b) Energy‐dispersive X‐ray mapping images of the PtMGs nanoparticles. Scale bar, 100 nm. c) UV–vis–NIR absorbance spectra of different nanocomposites (MOFs, PtMs, and PtMGs). d) XRD patterns of different nanocomposites (MOFs, PtMs, and PtMGs). e) Size distribution of PtMGs and PtMGs@HGd nanoparticles characterized by DLS. f) CD spectra of pure HSA and PtMGs@HGd. g) UV–vis spectra of free ICG, PtMGs@HGd, and ICG‐PtMGs@HGd. h) digital photograph of free ICG, PtMGs@HGd, and ICG‐PtMGs@HGd. i) Loading capacity under different ICG fed amounts. Data are presented as means ± SD (*n* = 3).

The octahedron porous gold nanoshells were fabricated using Pt encapsulated MOF as the inner template (Scheme 1). The Pt nanoparticles with the uniform sizes of around 3 nm were synthesized (**Figure** **1**a‐I), and were then redispersed in DMF, ZrCl_4,_ and the organic ligands (2‐aminoterephthalic acid (NH_2_‐BDC) to form amino modified Pt‐MOFs (PtMs).^[^ ^26^ ^]^ Transmission electron microscope (TEM) images showed that the Zr‐based MOFs maintained regular 3D octahedral structure, and the uniform size and dispersion after Pt doping (Figure 1a‐II,III), indicating the successful encapsulation of Pt into the as‐synthesized MOFs. Interestingly, the size of MOFs nanoparticles could be reduced after decoration with Pt NPs, because the introducing of Pt NPs can lead to the heterogeneous growth of the MOFs, increase the nucleation rate of the whole system and then result in the formation of smaller‐sized nanoparticles.^[^ ^27^ ^]^ This result was also confirmed by the dynamic light scattering (DLS) studies (Figure 1e), which showed the hydrodynamic diameter of MOFs and PtMs nanoparticles was 178.8 and 92.5 nm, respectively (both larger than the particle size of TEM data due to the hydrate particle size by Mastersizer DLS studies). An outerlayer of porous gold nanoshells was formed on the surface of the NH_2_‐modified PtMs (PtMGs) by anchoring the gold seeds from the overnight‐aged HAuCl_4_–K_2_CO_3_ solution to the amino groups on the surface of PtMs via electrostatic adsorption followed by local reduction in polyvinyl pyrrolidone (PVP) and a mild reductant (formaldehyde). This one‐step synthesis method^[^ ^21^ ^]^ enabled rapid, facile, and straightforward coating of the porous gold nanoshells outside the nanoparticle cores. TEM images revealed a relative uniform layer of gold nanoshells coated on the surfaces of PtMs and the morphological structures remained almost unchanged (Figure 1a‐IV). High‐angle annular dark‐field scanning TEM (STEM) and energy‐dispersive X‐ray (EDX) mapping of PtMGs further proved the formation of a uniform gold outerlayer and the encapsulation of Pt enzyme in the nanoparticles (Figure 1b). These results were further confirmed by the UV–vis–NIR spectra and the color change of the different formulations, which showed white color without significant peaks in the initial MOFs, while a brown color along with an obvious peak at 390 nm after Pt doping, and a blue‐green color along with a strong NIR absorption with the maximum value at about 800 nm after Au anchoring and reduction as a results of the characteristic surface plasmon resonance of the formed gold nanoshells (Figure 1c and Figure S1, Supporting Information). In addition, the similar powder X‐ray diffraction (XRD) profiles before and after Pt doping (Figure 1d) indicate the maintenance of the crystallinity and structural integrity of MOFs after Pt nanozyme loading. The remarkable increased intensity of Au diffraction peak (≈38°) in the XRD profile of PtMGs verified the formation of gold nanoshells (Figure 1d). We performed the experiment of Barrett‐Joyner‐Halenda model from adsorption branch of isotherms, and found that the pore size of the MOFs and PtMs were nearly the same (about 2.1 nm) (Figure S2, Supporting Information), indicating that the addition of Pt nanoparticles did not affect the pore diameter of the MOFs structure. What is more, the size of the as‐synthesized Pt nanoparticles (about 3 nm, Figure 1a‐I) was larger than the cavity size of the MOFs (about 2.1 nm, Figure S2, Supporting Information), supporting that the Pt nanoparticles did not occupy the porous structure of MOFs, but were wrapped by the surrounding MOFs. This result was in accordance with the previous studies showing that in the “bottom‐around‐ship” synthetic method, the hydrodynamic of radius of the metal nanoparticles was usually larger than the cavity size of the MOFs so that nanoparticles were wrapped by the surrounding MOFs rather than occupying the cavity of the MOFs.^[^ ^26^, ^28^ ^]^ The lower surface area of PtMs (810.88 m^2^ g^−1^) compared to the intrinsic MOFs (927.14 m^2^ g^−1^) was mainly due to the contribution of nonporous Pt nanoparticles and the existence of PVP in the composites.^[^ ^29^ ^]^ Taken together, these results demonstrated that the NH_2_‐modified Pt‐MOFs could serve as effective templates for the coating of the porous gold nanoshells by a facile and versatile method.

The obtained PtMGs were further decorated with HGd and loaded with ICG to establish the versatile PtMGs@HGd nanoparticles for the multimodal imaging‐guided cancer therapy. PtMGs nanoparticles were first decorated with 11‐mercaptoundecanoic acid (MUA) by Au—S bond to obtain PtMGs–COOH. After that, HSA‐Gd hybrids were covalently connected onto PtMGs‐COOH nanoparticles via carbodiimide‐catalyzed amidation reaction (Scheme 1). DLS characterization showed that the hydrodynamic diameter of the nanoparticles increased from 174.1 nm (PtMGs) to 203.5 nm (PtMGs@HGd) (Figure 1e) and the zeta potential of different formulations (PtMs, PtMGs, PtMGs‐COOH, and PtMGs@HGd) during the synthetic process was determined to be 34.5, ‐23.1, ‐42.8, and ‐15.6 mV, respectively (Figure S3, Supporting Information), suggesting the decoration of HGd on PtMGs. Circular dichroism (CD) (Figure 1f) shows that the typical bands of PtMGs@HGd were consistent well with the ones from the pure HSA, investigating the coating of HSA on the surface of the nanoplatform. However, the peak at ≈210 nm in the CD spectra of PtMGs@HGd showed a little blue shift compared with the CD spectra of the pure HSA, suggesting an increase of random coil structures after the decoration of HGd hybrids.^[^ ^30^ ^]^ The T_1_‐weighted MR imaging capacity and the corresponding signal intensity of ICG‐PtMGs@HGd exhibited a relatively high value among existing Gd‐based contrast agents^[^ ^31^ ^]^ (Figure S4a,b, Supporting Information), further confirming that the HGd hybrids was anchored to PtMGs@HGd. In addition, the molar ratio of Zr, Pt, Gd, and Au in PtMGs@HGd was detected to be 9.2, 3.2:1.8:100 by ICP‐MS, guaranteeing the H_2_O_2_ catalytic effect and MR imaging of the nanoplatform owing to the abundant loading content of Pt and Gd.

ICG molecule could be loaded into the PtMGs@HGd via being encapsulated into the porous gold nanoshells or via binding with HSA through the hydrophobic interaction between the small molecule and the hydrophobic domain of protein.^[^ ^32^ ^]^ In the UV–vis spectra, the absorption of ICG‐PtMGs@HGd showed a characteristic peak at 800 nm with a slight red shift and a wider absorption compared with the free ICG (Figure 1g), indicating the existence of ICG in the nanoplatform. What is more, the external color changes of the PtMGs@HGd solution from blue to green also indicated the successful loading of ICG (Figure 1h). The measured loading capacity of ICG in the PtMGs@HGd nanoparticles (weight ratio between ICG and ICG‐PtMGs@HGd) increased along with the raise of the fed amount of ICG and finally reached a maximum of 26.0% at an ICG amount of 400 µg (Figure 1i). Moreover, there were no obvious differences in the ICG release profile at different pH (pH 5.0, 6.5, and 7.4) (Figure S5, Supporting Information), indicating that the pH value barely affected the release behavior of the nanoparticles.

In addition, we also evaluated the stability of ICG‐PtMGs@HGd in different media (deionized water, PBS, cell culture medium of DMEM and RPMI‐1640 with 10% fetal bovine serum (FBS)). Visual photographs of the samples after 72 h showed that the ICG‐PtMGs@HGd nanoparticles were well dispersed without aggregation in all the media (Figure S6a, Supporting Information). We also measured the particles sizes of the samples in different media at different time points (0, 24, 48, and 72 h) by DLS, and observed no obvious changes in all the groups over 72 h (Figure S6b, Supporting Information), indicating the high stability of the nanocarriers in different physiological solutions.

### 2.2 Catalase Activity Mediated Improved PDT Effect In Vitro

**Figure 2:**
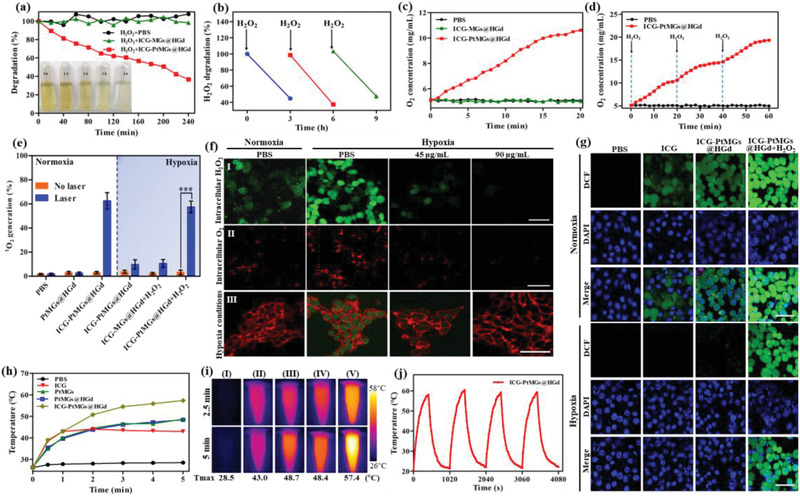
Improved PDT and PTT effect of ICG‐PtMGs@HGd. a) H_2_O_2_ degradation after treated with PBS and ICG‐PtMGs@HGd at different time points. Inset: the corresponding color changes of Ti (SO_4_)_2_ solution treated with ICG‐PtMGs@HGd. b) Continuous H_2_O_2_ consumption after treated with ICG‐PtMGs@HGd nanoparticles by repetitive addition of H_2_O_2_. c) O_2_ generation after treated with PBS, ICG‐MGs@HGd, and ICG‐PtMGs@HGd nanoparticles at predesigned time points. d) Repetitive O_2_ generation after treated with ICG‐PtMGs@HGd by repeated addition of H_2_O_2_. e) The ^1^O_2_ generation efficiency of different formulations determined by DPBF under normoxia and hypoxia conditions, data are presented as means ± SD (*n* = 3). ***P < 0.001 (Student's t‐test). f) CLSM fluorescent images showing intracellular H_2_O_2_ consumption (I), O_2_ generation (II) and cellular hypoxic conditions (III) of 4T1 cells treated with PBS and ICG‐PtMGs@HGd nanoparticles (45 and 90 µg mL^−1^) under the normoxia and hypoxia conditions. MAK 164, (Ru(dpp)_3_) Cl_2_, Alexa Fluor 488‐conjugated HIF‐1*α*, and rhodamine‐conjugated phalloidin were used as H_2_O_2_, O_2_, hypoxia and tubulin indicator, respectively. Scale bar, 50 µm (I) 200 µm (II) and 50 µm (III). g) CLSM images of 4T1 cells treated with PBS, ICG, ICG‐PtMGs@HGd, and ICG‐PtMGs@HGd + H_2_O_2_ with laser irradiation under the normoxia and hypoxia conditions for ROS detection (laser: 1.5 W cm^−2^, 808 nm, 5 min). Scale bar, 50 µm. h) Laser‐triggered temperature elevation curves and i) thermographic images of different formulations of PBS (I), ICG (II), PtMGs (III), PtMGs@HGd (IV) and ICG‐PtMGs@HGd (V) over a period of 5 min's laser irradiation (808 nm, 1.5 W cm^−2^). j) Photothermal curve of ICG‐PtMGs@HGd for four repeated irradiation cycles.

The catalase‐like activity of ICG‐PtMGs@HGd was investigated by time‐dependent H_2_O_2_ consumption assay using titanium sulfate (Ti(SO4)_2_) as a colorimetric indicator.^[^ ^33^ ^]^ The UV–vis absorption spectra of H_2_O_2_–Ti(SO_4_)_2_ decreased with time after the treatment with ICG‐PtMGs@HGd and the calculated degradation ratio of H_2_O_2_ showed that about 70% of H_2_O_2_ was consumed within 240 min after the addition of ICG‐PtMGs@HGd (**Figure** **2**a and Figure S7, Supporting Information). By comparison, PBS and ICG‐MGs@HGd groups showed no obvious catalysis of H_2_O_2_ ((Figure 2a). These results indicated the efficient catalytic activity of ICG‐PtMGs@HGd. We measured the generation of O_2_ with the dissolved oxygen meter and found that the O_2_ concentration of ICG‐PtMGs@HGd solution rapidly increased from 5.0 to 10.8 mg L^−1^ after the introduction of H_2_O_2_, whereas no notable oxygen production was observed in the absence of Pt nanomaterials (Figure 2c), further demonstrating the Pt‐induced catalytic effect of ICG‐PtMGs@HGd. Pt nanozyme has been known to persistently produce O_2_ via catalyzing H_2_O_2_ that is continuously generated by the hypoxic tumor cells.^[^ ^15^ ^]^ We therefore detected the degradation of H_2_O_2_ and the generation of O_2_ after repetitive addition of H_2_O_2_ that was used to mimic the in vivo microenvironment of tumor. As expected, ICG‐PtMGs@HGd nanoparticles retained nearly undecayed consumption of H_2_O_2_ and stable production of O_2_ (Figure 2b,d), indicating the persistent catalytic capacity of the nanocarriers.

After confirming the effective H_2_O_2_ catalytic capacity of ICG‐PtMGs@HGd, we further tested whether ICG‐PtMGs@HGd could serve as an oxygen supplier for the enhanced PDT effect at a mimetic H_2_O_2_ and hypoxia environment. The generation of ^1^O_2_ was measured using 1,3‐diphenylisobenzonfuran (DPBF) as an probe.^[^ ^34^ ^]^ As seen in Figure 2e, under hypoxic condition, there was negligible production of ^1^O_2_ in PBS and PtMGs@HGd solutions after light irradiation, while the production of ^1^O_2_ significantly increased when ICG was loaded (ICG‐PtMGs@HGd), demonstrating the PDT effect of ICG, which was in accordance with the previous reports showing that ICG could not only serve as an NIR dye for fluorescent and photoacoustic imaging, but could also generate ROS and hyperthermia for PDT and PTT. The generation of ^1^O_2_ from ICG‐PtMGs@HGd after light irradiation significantly deceased under hypoxic condition, but could be obviously enhanced when adding H_2_O_2_. However, this H_2_O_2_‐induced enhancement of ^1^O_2_ production was not observed in the ICG‐MGs@HGd nanoparticles without Pt nanozyme, further verifying the Pt nanozyme‐induced catalytic activity and the resulting improved PDT effect of ICG‐PtMGs@HGd under hypoxic atmosphere and mimetic H_2_O_2_ environment.

We then investigated the catalase activity and PDT effect of ICG‐PtMGs@HGd at the cellular level. H_2_O_2_‐pretreated 4T1 cells were incubated with the ICG‐PtMGs@HGd dispersion at low (45 µg mL^−1^) and high (90 µg mL^−1^) concentrations, and MAK164, an H_2_O_2_ probe, was used to estimate the decomposition of intracellular H_2_O_2_.^[^ ^21^ ^]^ We found that the fluorescent intensity of the cells significantly decreased in a concentration‐dependent trend after incubation with ICG‐PtMGs@HGd (Figure 2f‐I), indicating that ICG‐PtMGs@HGd nanoparticles could successfully catalyze the intracellular H_2_O_2_. We further investigated the capability of ICG‐PtMGs@HGd to alleviate hypoxia by assessing the endogenous O_2_ generation using an intracellular O_2_ indicator [Ru(dpp)_3_]Cl_2_ whose fluorescence could be quenched after reacting with O_2_ molecule.^[^ ^35^ ^]^ 4T1 Cells treated with ICG‐PtMGs@HGd exhibited decayed red fluorescence in a concentration‐dependent manner compared with the ones without ICG‐PtMGs@HGd treatment under hypoxic condition (Figure 2f‐II), confirming the remarkable intracellular O_2_ generation via Pt‐induced catalytic reaction and the subsequently successful modulation of the intracellular hypoxic milieu. Moreover, the expression level of hypoxia‐inducible factor‐1*α* (HIF‐1*α*) was used as the hypoxia indicator to evaluate the catalytic activity of ICG‐PtMGs@HGd since the hypoxic cells are characterized by intracellular accumulation of HIF‐1*α*.^[^ ^36^ ^]^ We used anti‐HIF‐1*α* antibody and rhodamine‐conjugated phalloidin to costain HIF‐1*α* and tubulin in 4T1 cells under normoxia and hypoxia conditions. As expected, the cells under normoxic condition showed no expression of HIF‐1*α* (green), while the cells under hypoxic condition exhibited significant accumulation of HIF‐1*α* (Figure 2f‐III). The immunofluorescence of HIF‐1*α* significantly decreased after incubation with ICG‐PtMGs@HGd (Figure 2f‐III), further confirming ICG‐PtMGs@HGd‐mediated oxygen generation under hypoxic condition. These results demonstrated the capability of ICG‐PtMGs@HGd in decomposing H_2_O_2_ to produce persistent oxygen under hypoxic conditions for efficient PDT effect.

ROS generation in the tumor cells under the laser irradiation was investigated by fluorescent imaging with the ROS indicator DCFH‐DA.^[^ ^37^ ^]^ As expected, both free ICG and ICG‐PtMGs@HGd showed obvious ROS production under normoxia condition. Nearly no fluorescence was observed with ICG and ICG‐PtMGs@HGd under hypoxia condition, but significant fluorescence was observed with ICG‐PtMGs@HGd when H_2_O_2_ was added (Figure 2g), indicating ROS generation mediated by the catalytic activity of ICG‐PtMGs@HGd.

All of these results demonstrated the Pt‐doping nanoparticles possessed continuously stable catalase‐like activity and could generate O_2_ to improve PDT efficacy under the hypoxia environment in the tumor cells, broadening an avenue for the application of metal materials‐based nanozyme in the biomedical field.

### 2.3 PTT Effect of ICG‐PtMGs@HGd In Vitro

We then explored the photothermal performance of ICG‐PtMGs@HGd. Exposed to laser irradiation for 5 min, PtMGs@HGd exhibited a concentration‐ and irradiation time‐dependent temperature increased characteristics (Figure S8, Supporting Information). We also performed the DLS experiment of the PtMGs@HGd nanocarriers before and after laser irradiation, and found their size distribution and zeta potential kept well after laser irradiation (Figure S9, Supporting Information), suggesting the favorable stability of the nanoparticles when irradiated by laser. We next compared the PTT effect of different formulations, and found that ICG‐PtMGs@HGd possessed significantly higher thermal properties compared to PtMGs@HGd and free ICG (Figure 2h), which was also confirmed by the real‐time variations of temperature of different formulations recorded by infrared thermal imaging camera (Figure 2i). These results, again, demonstrated the PTT effect of ICG, and also revealed the improvement of PTT effect by the synergistic therapy. We also observed that the temperature elevation of PtMGs and PtMGs@HGd were nearly the same (Figure 2h,i), suggesting that the PTT effect mainly derived from the inner gold nanoshells rather than from the outside HSA–Gd complexes. Importantly, the increased temperature of ICG‐PtMGs@HGd kept nearly unchanged after four repeated cycles of laser irradiation (Figure 2j), indicating the excellent photothermal stability of the nanoplatform. The photothermal conversion efficiency (*η*) of the ICG‐PtMGs@HGd nanoparticles was determined to be 38.55% (Figure S10, Supporting Information), which was higher than the conventional gold nanoshells.^[^ ^1^, ^4^ ^]^ This may be attributed to the unique octahedral structure of the MOF‐based nanoparticles in which the multiple sharp points can effectively make the nanoparticles capture more laser energy.^[^ ^38^ ^]^

We further investigated whether photothermal effect can enhance the Pt catalyzed O_2_ generation during therapeutic treatment. As shown in Figure S11 (Supporting Information), the increased oxygen of PtMGs@HGd (from 4.64 to 11.55 mg mL^−1^) and PtMGs@HGd + Laser (from 4.80 to 11.41 mg mL^−1^) had no obvious difference, demonstrating the stability of Pt‐catalyzed effect is excellent and the oxygen generation could not be affected by the photothermal effect.

### 2.4 Cellular Uptake and Synergistic Phototherapy

**Figure 3:**
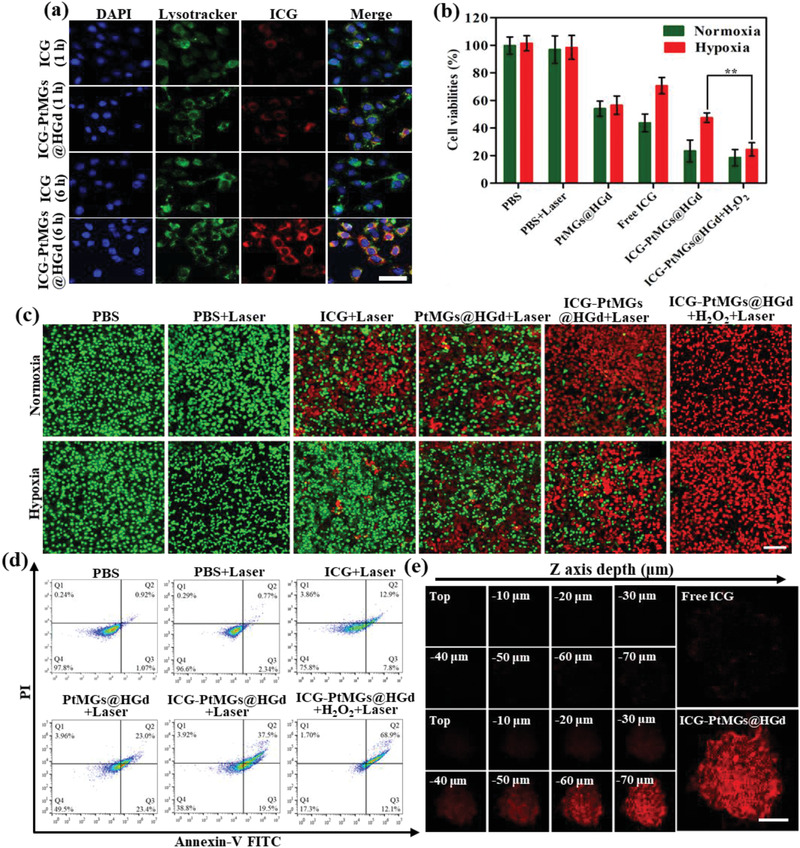
In vitro evaluation of the enhanced antitumor effect of ICG‐PtMGs@HGd. a) CLSM images of 4T1cells incubated with free ICG and ICG‐PtMGs@HGd for 1 or 6 h, respectively. Scale bar, 50 µm. b) Cytotoxicity of different formulations (PBS, PBS + Laser, ICG + Laser, PtMGs@HGd + Laser, ICG‐PtMGs@HGd + Laser and ICG PtMGs@HGd + H_2_O_2_ + Laser, respectively) incubated with 4T1 cells under normoxia and hypoxia conditions. (Laser: 1.5 W cm^−2^, 808 nm, 5 min). Data are presented as means ± SD (*n* = 3). **P < 0.01 (Student's t‐test). c) Live/dead cell assay for 4T1 cells treated with different formulations under normoxia and hypoxia conditions (green: live cells, red: dead cells). (Laser: 1.5 W cm^−2^, 808 nm, 5 min). Scale bar, 100 µm. d) Flow cytometry analysis of FITC‐annexin V/PI double‐stained 4T1 cells after treated with various formulations under a hypoxic condition. e) Representative Z‐stack scanning confocal images of ICG (upper lane) and ICG‐PtMGs@HGd (lower lane) penetrated 4T1 tumor spheroids. (Laser: 1.5 W cm^−2^, 808 nm, 5 min). Scale bar, 100 µm.

The cellular uptake and the endocytosis mechanism of ICG‐PtMGs@HGd were investigated. As it has been widely believed that the cellular endocytosis of the nanoparticles is closely associated with the endolysosomal compartments,^[^ ^39^ ^]^ we utilized LysoTracker Green, a lysosome binding dye,^[^ ^4c^ ^]^ to ascertain the localization of the lysosomes and the nanocarriers. Confocal microscopy images showed a well colocalization of the fluorescence of ICG (red) and LysoTracker (green) after incubating of ICG‐PtMGs@HGd with the murine breast cancer cell line 4T1 cells for 1 h (**Figure** **3**a). The Pearson's correlation coefficient representing the colocalization of ICG fluorescence of ICG‐PtMGs@HGd nanoparticles and LysoTracker was determined to be 0.89 (Figure S12, Supporting Information), suggesting that ICG‐PtMGs@HGd was internalized by the tumor cells via the endolysosomal pathway.^[^ ^40^ ^]^ The stronger fluorescence of ICG in ICG‐PtMGs@HGd compared to free ICG was due to the improved cellular uptake activity of ICG‐PtMGs@HGd nanoparticles. After 6 h incubation, the fluorescence of ICG became stronger and gradually separated with LysoTracker (Pearson's correlation of 0.48, Figure S12, Supporting Information), indicating that ICG‐PtMGs@HGd could effectively achieve endosome/lysosome escape after cellular uptake, which is a critical prerequisite for cancer therapy.^[^ ^40^ ^]^ In contrast, nearly no fluorescence was observed with free ICG after 6 h incubation, which might be attributed to the reduced degradation of ICG after loading into the PtMGs@HGd nanocarriers.

We then investigated the synergistic phototherapy effect of ICG‐PtMGs@HGd in vitro. MTS assay showed that without laser irradiation, the viability of 4T1 cells maintained above 90% even when incubated with the highest concentration of ICG‐PtMGs@HGd (ICG: 40 µg mL^−1^, PtMGs@HGd: 320 µg mL^−1^) (Figure S13, Supporting Information), indicating that ICG‐PtMGs@HGd did not induce apparent cytotoxicity and had excellent biocompatibility. We then quantitatively elucidated the photoinduced antitumor efficiency of the various preparations. As shown in Figure 3b, under normoxia condition, both PtMGs@HGd and free ICG displayed a relative strong cancer cell killing effect (cell viability of 54.05% ± 5.49% and 43.71% ± 6.48%, respectively), while ICG‐PtMGs@HGd significantly enhanced the cell lethality (cell viability of 23.30% ± 7.90%), indicating improved therapeutic efficiency due to the combination of ICG and PtMGs@HGd. By comparison, under hypoxia condition, the toxicity of free ICG and ICG‐PtMGs@HGd to 4T1 cells obviously decreased as a result of a less ^1^O_2_ production in PDT effect derived from the insufficient oxygen, but could be significantly improved when adding H_2_O_2_. This could be attributed to the elevated continuous O_2_ supplementation via the catalyze effect of Pt enzyme that was encapsulated in ICG‐PtMGs@HGd. These results were further confirmed by fluorescence costaining using calcein‐AM/PI to visualize the live (green) and the dead (red) cells, respectively. Consistent with the findings in MTS assay, we observed that under normoxia condition, the apoptosis of 4T1 cells was significantly higher when treated with ICG‐PtMGs@HGd than treated with free ICG or PtMGs@HGd (Figure 3c), indicating the enhanced antitumor effect due to the synergistic treatment. Decreased red fluorescence was observed in the ICG‐PtMGs@HGd group under hypoxic condition (Figure 3c) confirming that the PDT efficiency was affected by the hypoxia. This hypoxia‐induced decline of the antitumor effect was obviously eased when an abundant of O_2_ was provided by the catalysis of ICG‐PtMGs@HGd under the H_2_O_2_‐containing environment (Figure 3c). The apoptosis and necrosis assay under hypoxic condition was further performed by flow cytometry with Annexin V‐FITC/PI kit to investigate the level and the mechanism of cell death^[^ ^24a^ ^]^ (Figure 3d). A cell survival of 75.8% was detected when the 4T1 cells were treated with free ICG, suggesting slight apoptosis. After combing ICG with the PtMGs@HGd nanoparticles, the joint treatment significantly increased the antitumor efficacy and the survival ratio of 4T1 decreased to about 39%. The antitumor effect significantly increased when H_2_O_2_ was added (ICG‐PtMGs@HGd + H_2_O_2_ group) and a cell survival rate of 17.3% was achieved, demonstrating enhanced PDT effect by Pt catalysis‐induced O_2_ generation. These results together with the in vitro cytotoxicity assay and the live/dead staining assay, demonstrated that ICG‐PtMGs@HGd nanoparticles could effectively kill tumor cells by synergistic O_2_‐evolving PDT/PTT effect to overcome hypoxic tumor environment. Tumor‐associated fibroblast cells and extracellular matrix in the TME, which contains collagen, glycosaminoglycans, and proteoglycans, have become a primary barrier for diffusion of multifunctional nanoparticles,^[^ ^41^ ^]^ so the deep penetration ability of the nanomaterials is vitally important for cancer therapy. Here the penetration ability of ICG‐PtMGs@HGd was investigated via 3D spheroids tumor model, which can mimic the pathological penetration barrier of nanoparticles in the solid tumor.^[^ ^42^ ^]^ The Z‐stack scanning exhibited stronger fluorescence signals in the ICG‐PtMGs@HGd group compared with free ICG (Figure 3e), indicating deeper penetration of the ICG molecules after being loaded into the PtMGs@HGd nanoparticles. This might be due to the increased stability of ICG after loading into the nanoparticles as ICG tends to aggregate and degrade rapidly in the aqueous medium. Thus nanoparticle encapsulating would greatly decrease the degradation rate and therefore prolonged the short plasma half‐life of ICG.^[^ ^43^ ^]^

### 2.5 Florescence/MSOT/CT/MR Quad‐Modal Imaging In Vivo

**Figure 4:**
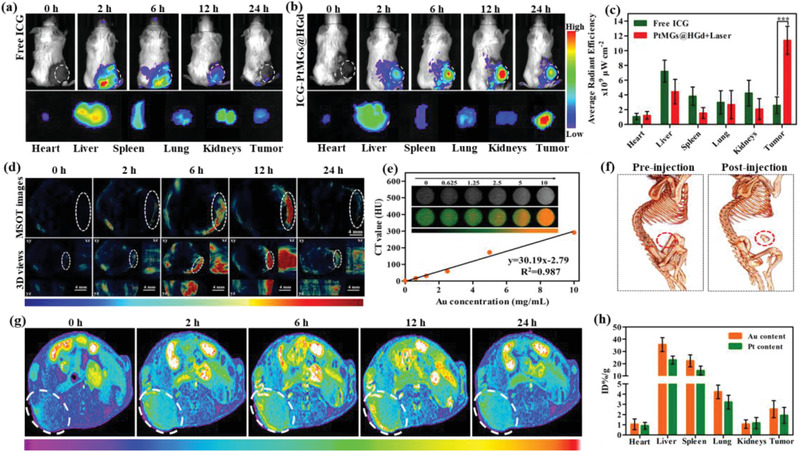
In vivo multimodal imaging. a) Real‐time fluorescent images of 4T1 tumor‐bearing mice at different time points after administration of free ICG and b) ICG‐PtMGs@HGd; the bottom panel shows the ex vivo images examined at 24 h postinjection. c) ICG fluorescent intensities from ex vivo imaging of the major organs and the tumors 24 h after injecting free ICG and ICG‐PtMGs@HGd. Data are presented as mean ± SD. ***P < 0.001 (Student's t‐test). d) The MSOT and 3D orthogonal MSOT images of 4T1 tumor‐bearing mice after being intravenously injected with ICG‐PtMGs@HGd nanoparticles at different time points. e) CT signal intensity linearly fits to the concentration of ICG‐PtMGs@HGd aqueous solutions; inset: the corresponding CT images. f) 3D reconstructed CT images before and 12 h after injection of ICG‐PtMGs@HGd nanoparticles. g) T_1_‐weighted MR images after intravenous injection with ICG‐PtMGs@HGd at predesigned time points. h) The body distribution of Au and Pt content 24 h after the intravenous injection of ICG‐PtMGs@HGd nanoparticles.

Nanoplatform that supply complementary anatomical and functional information about tumors by multimodal imaging is of great importance for the precise detection and treatment of cancer. We therefore evaluated the multimodal imaging capacity of the multifunctional ICG‐PtMGs@HGd nanoplatform in 4T1 tumor‐bearing mice. As the fluorescence of ICG enabled it a desired agent for fluorescent imaging, we first checked the fluorescent imaging capacity of the nanoparticles. We measured the time‐dependent biodistribution of free ICG and ICG‐PtMGs@HGd in 4T1 tumor‐bearing mice at pre‐designed time points (0, 2, 6, 12, and 24 h, respectively) after intravenous injection of the nanoparticles, and found that the fluorescence in the tumor region gradually increased over time in both free ICG or ICG‐PtMGs@HGd injected mice (**Figure** **4**a,b). The fluorescence of free ICG accumulated at the tumor sites 2 h after injection and gradually diminished with time until 24 h when the fluorescence was dramatically decayed (Figure 4a). In comparison, with the fluorescence of ICG‐PtMGs@HGd, largely accumulated in the tumor 12 h after injection and remained detectable after 24 h (Figure 4b), suggesting that ICG‐PtMGs@HGd could triumphantly accumulate at tumors and retain for a long period of time. The ex vivo results showed tumors of the ICG‐PtMGs@HGd treated mice had a stronger fluorescence signal than the metabolic organs, such as the liver, spleen and kidney (Figure 4a–c), suggesting the high tumor accumulation of ICG‐PtMGs@HGd nanoparticles.

Since the high tumor uptake of ICG‐PtMGs@HGd by fluorescence imaging, we expected that these nanocarriers would also have outstanding contrast MSOT imaging owing to their strong capability in NIR absorption and photothermal conversion. We first measured the multispectral optoacoustic intensity of ICG‐PtMGs@HGd at different concentrations of ICG, and found that the MSOT signals increased linearly with ICG concentration (Figure S14, Supporting Information), suggesting the ICG‐induced capability of MSOT imaging in ICG‐PtMGs@HGd. We then evaluated the capability of ICG‐PtMGs@HGd for MSOT imaging in vivo by acquiring MSOT and 3D orthogonal MSOT images of the tumors at various time points post intravenous injection. We found that the MSOT signals of ICG‐PtMGs@HGd appeared at the tumor margin 2 h postinjection, and then gradually increased over time and achieved the maximum at 12 h postinjection (Figure 4d), demonstrating the capacity of ICG‐PtMGs@HGd for long duration MOST imaging and effective tumor homing.

Inspired by the view that the diagnostic advantages of ICG‐PtMGs@HGd could be improved by complementing the anatomical imaging modality with the functional imaging modality, we also investigated CT imaging of the nanoplatform owing to its high attenuation of X‐rays by Au atom.^[^ ^44^ ^]^ The CT contrast of the ICG‐PtMGs@HGd dispersions was investigated by phantom images in vitro at different Au concentrations (Figure 4e). We found that the CT images became gradually brighter and showed a superior linear relationship along with the increasing Au concentrations. To confirm the in vivo CT imaging, 4T1 tumor‐bearing mice were directly injected with the ICG‐PtMGs@HGd and then imaged by a small animal X‐ray CT imaging system. An obvious tumor contrast was detected after 12 h injection (Figure 4f), verifying the predominant CT imaging capacity of ICG‐PtMGs@HGd.

Since MRI can supply clear information of the soft tissue structure without ionizing radiation,^[^ ^45^ ^]^ we also investigated the MRI performance of ICG‐PtMGs@HGd using gadopentetic acid (Gd‐DTPA),^[^ ^31^, ^46^ ^]^ a clinically used MRI contrast agent, as the control. As expected, the in vitro T_1_‐weight MR images showed that the MRI signals from both ICG‐PtMGs@HGd and Gd‐DTPA significantly increased with Gd concentration, and a linear correlation was found between the longitudinal relaxation rate (1/*T*_1_) of water protons and the concentration of Gd (III) (Figure S4a,b, Supporting Information), confirming the effective MRI performance of Gd (III). The MRI signal from ICG‐PtMGs@HGd was much higher than the one from Gd‐DTPA at each concentration of Gd (Figure S4a,b, Supporting Information), indicating better MRI performance of ICG‐PtMGs@HGd compared to Gd‐DTPA. The molar relaxivity (*r*_1_),^[^ ^31^, ^46^, ^47^ ^]^ an important parameter that determines the efficiency of an MRI contrast agent, was calculated based on the slope of the line representing the linear correlation between 1/*T*_1_ and the concentration of Gd (III). As shown in Figure S4b (Supporting Information), the *r*_1_ value of ICG‐PtMGs@HGd nanoparticles was 35.69 mm ^−1^ s^−1^, 2.2‐fold higher than that of Gd‐DTPA (16.52 mm ^−1^ s^−1^), further demonstrating the excellent Gd‐mediated MRI capacity of this nanoparticle. A clear outline of the tumor was delineated in the in vivo T_1_‐weight MR images 12 h postintravenous injection when the brightest image with a sharp border was achieved (Figure 4g), and the relative quantitative values (Figure S15, Supporting Information) at different time points also confirmed the strongest signals after 12 h intravenous injection of the nanoparticles. The results suggested the good MRI performance and efficient tumor accumulation of ICG‐PtMGs@HGd.

Taken together, these data verified that ICG‐PtMGs@HGd could be used as a versatile agent for fluorescence/MSOT/CT/MR quad‐modal imaging with high contrast, and could provide more information about tumor from a broader spectrum.

### 2.6 Biodistribution and Blood Circulation of ICG‐PtMGs@HGd In Vivo

We quantitatively investigated the biodistribution of ICG‐PtMGs@HGd in mice by determine the Au and Pt content in the tumors and major organs, including the heart, liver, spleen, lung, and kidney, based on the percentage of injected dose per tissue gram (% ID/g) via ICP‐MS spectrometry (Figure 4h). After intravenous injection of the nanomaterials for 24 h, large amount of Au and Pt element appeared in liver and spleen due to the reticuloendothelial system (RES) clearance. Around 2.5% of Au and 1.9% of Pt of the nanoparticles accumulated in the tumor, consistent with the previous reports.^[^ ^48^ ^]^ To evaluate the blood circulation behavior, blood samples were collected at designed time points and then Au concentration were detected by ICP. The blood circulation curve showed the ICG‐PtMGs@HGd nanoparticles were gradually metabolized and the half‐time of blood circulation was 2.52 ± 0.23 h (Figure S16, Supporting Information).

### 2.7 Synergistic Phototherapy In Vivo

**Figure 5:**
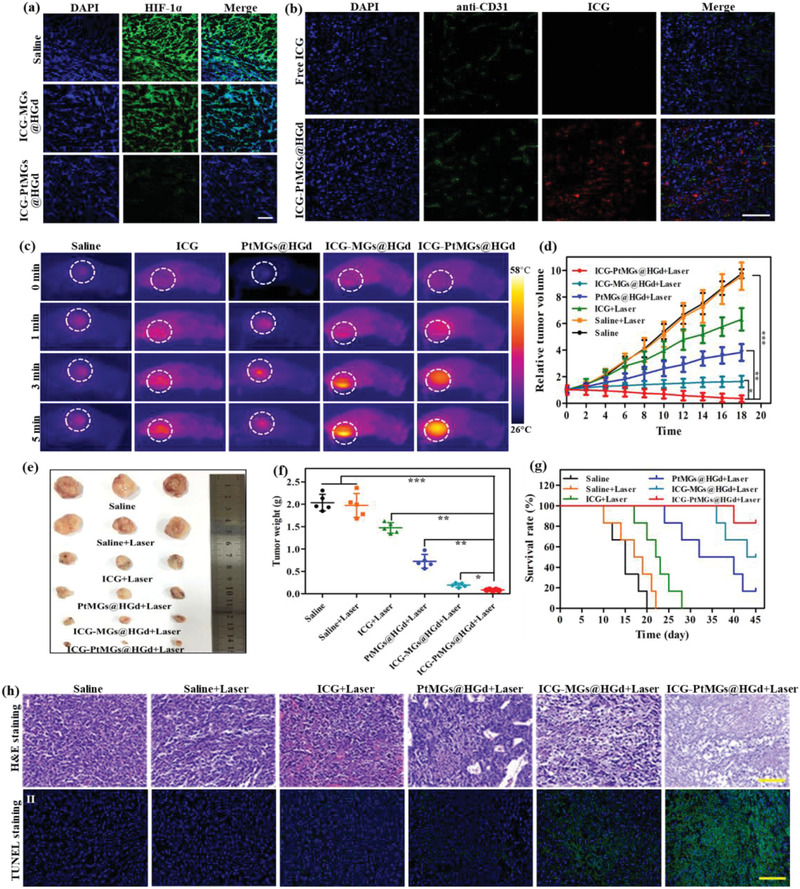
In vivo synergistic phototherapy in 4T1 tumor‐bearing mice. a) Representative immunofluorescent images showing the hypoxia staining of tumor slices after different treatments. The hypoxia areas were stained anti‐HIF‐1*α* antibody. Scale bar, 50 µm. b) Intratumoral distribution of ICG‐PtMGs@HGd nanoparticles. The nuclei were stained with DAPI (blue), and the tumor blood vessels were stained with FITC‐labeled CD31 antibody (green). Scale bar, 50 µm. c) Infrared thermographic images of 4T1 tumor‐bearing mice exposed to an 808 nm laser (1.5 W cm^−2^, 5 min) for determined time intervals 12 h postintravenous injection of different formulations. d–g) The tumor growth curves, representative photographs of the excised tumors, the excised tumor weights and the survival rates of 4T1 tumor‐bearing mice after different treatments. *P < 0.05; **P < 0.01; ***P < 0.001 (Student's t‐test). h) H&E staining (I) and TUNEL staining (II) of the tumor tissues after various treatments. Scale bar, 100 µm.

Inspired by the excellent Pt enzyme‐induced catalase activity under hypoxia and the high phototherapeutic efficacy of ICG‐PtMGs@HGd as was proved in vitro, we further explored the antitumor efficacy of ICG‐PtMGs@HGd in vivo. We first investigated if ICG‐PtMGs@HGd could increase the oxygen content and therefore conquer the hypoxia environment of the tumor. The tumor slices were stained with the antibody specific to hypoxia‐inducible factor 1*α* (HIF‐1*α*), a protein that has been reported to be upregulated under the hypoxic condition,^[^ ^49^ ^]^ to track the hypoxia area. Immunofluorescent staining showed that the tumor treated with ICG‐PtMGs@HGd exhibited significant lower content of HIF‐1*α* compared to the one treated with saline or ICG‐MGs@HGd (**Figure** **5**a), suggesting that the hypoxia of the tumor tissue was successfully alleviated due to the catalytic effect of Pt enzyme. The intratumoral distribution of ICG‐PtMGs@HGd was also evaluated. Obvious, a greater ICG fluorescent signal was observed in ICG‐PtMGs@HGd compared to free ICG (Figure 5b), indicating increased capacity of ICG in the tumor after loaded into the nanocarrier. We further characterized the microvessels with anti‐CD31, which can specifically react with CD31, a marker protein in the vascular endothelial cells,^[^ ^50^ ^]^ and found that the fluorescence of ICG was not completely colocalized with the one of the tumor microvessels (Figure 5b), implying that ICG‐PtMGs@HGd could extravasate from the microvessels and penetrate into the deeper part of the tumors, and would therefore achieve a better phototherapeutic efficacy.

We then investigated the photothermal effect of ICG‐PtMGs@HGd in the tumor‐bearing mice by the infrared thermal camera, which revealed that all the nanoparticles (free ICG, MGs@HGd, ICG‐MGs@HGd, and ICG‐PtMGs@HGd) exhibited the temperature elevation with increasing irradiation time compared to the negligible temperature elevation in the PBS group (Figure 5c and Figure S17, Supporting Information). The temperature elevation was significant (from 37.6 °C to 55.8 °C) in the synergistic formulations (ICG‐MGs@HGd and ICG‐PtMGs@HGd, with the same Au concentration) but was moderate in the solo therapies (free ICG and PtMGs@HGd) (Figure 5c and Figure S17, Supporting Information), indicating better photothermal performance of the combined therapy. No difference of the temperature elevation was found in ICG‐MGs@HGd and ICG‐PtMGs@HGd (Figure 5c and Figure S17, Supporting Information), indicating that Pt had no effect on PTT effect. To further assess the antitumor efficacy of the laser‐triggered combined PTT/Oxy‐PDT effect, the 4T1 tumor‐bearing mice were intravenously injected with different formulations and were irradiated with the NIR laser 12 h postinjection. The tumor sizes and body weights were measured every other day. On day 18, the mice from different groups were sacrificed, then the tumors were collected and further weighed. Encouragingly, ICG‐PtMGs@HGd treatment led to the most significant elimination of the tumor volume (Figure 5e), and the obtained representative photos of isolated tumors from each group (Figure 5d and Figure S18, Supporting Information) were further displayed. The lowest tumor weight (Figure 5f) compared to the free ICG, PtMGs@HGd and ICG‐MGs@HGd group also confirmed improved antitumor effect by Pt catalysis‐assisted synergistic phototherapy. We also monitored the survive rate of the tumor‐bearing mice under different treatments. As expected, the control group exhibited early death, while the treatment with ICG, PtMGs@HGd, and ICG‐MGs@HGd moderately prolonged the survival time. ICG‐PtMGs@HGd performed the best antitumor effect with the overall survival rate of 83% after 45 d (Figure 5g), again demonstrating the improved antitumor effect of ICG‐PtMGs@HGd. Moreover, the body weights of the tumor‐bearing mice after all the treatments showed no obvious changes (Figure S19, Supporting Information), indicating the low toxicity of the different formulations. H&E and TUNEL staining of the tumor tissue affirmed the most extensive apoptosis and necrosis in the ICG‐PtMGs@HGd + laser group (Figure 5h), demonstrating the improved capacity of the synergistic phototherapy formulation to inhibit tumor growth.

### 2.8 Inhibition of Tumor Metastasis

**Figure 6:**
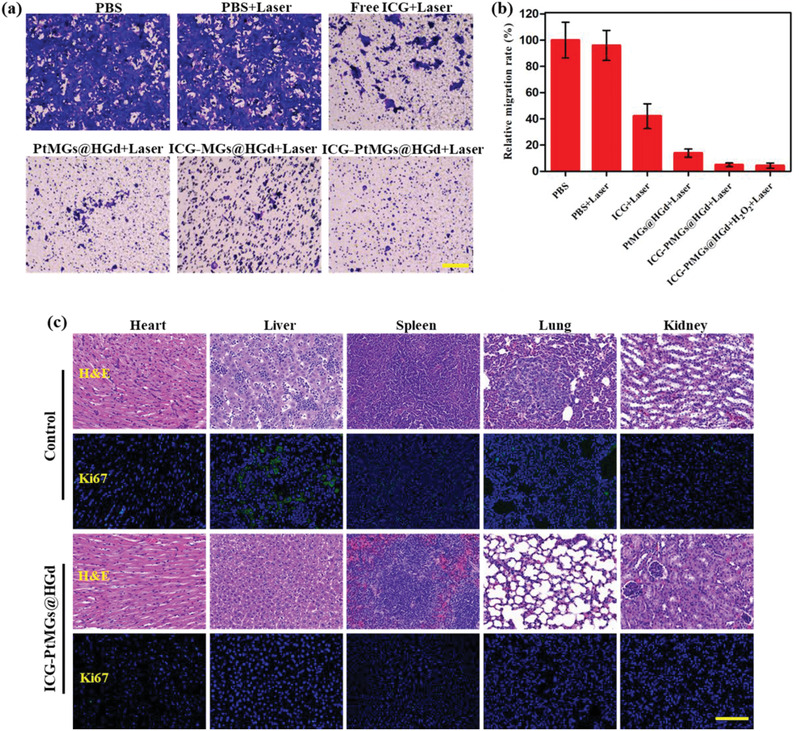
Inhibition of tumor metastasis. a) Representative micrographs of 4T1 cells passed through the transwell membrane after treated with different formulations. Scale bar, 100 µm. b) Relative migration rates of 4T1 cells after different treatment calculated from Figure 6a. Data are presented as mean ± SD. c) Representative H&E and Ki‐67 immunostaining photomicrographs of the major organ tissues. Scale bar, 100 µm.

4T1 tumors were known to be highly malignant and to have strong metastatic tendency to lungs and many other organs.^[^ ^51^ ^]^ Thus, we also investigated the effect of ICG‐PtMGs@HGd to inhibit tumor metastasis in vitro and in vivo. As shown in **Figure** **6**a,b, inhibited migration of 4T1 cells through the transwell membrane was detected after incubated with the nanoparticles. By comparison, the cells treated with PBS exhibited serious cell migration. Among all the treatments with the nanoparticles, ICG‐PtMGs@HGd + H_2_O_2_ showed the lowest cell migration, indicating enhanced inhibition of the cell migration by the synergistic phototherapy. The tumors and the major organs of the tumor‐bearing mice after treatment were further collected for H&E and Ki‐67 immunostaining to investigate the extent of tumor metastasis in vivo. H&E staining showed that saline group possessed clear metastatic sites in the lung and the liver, which were characteristic with the infiltrated tumor cells (Figure 6c).^[^ ^52^ ^]^ As the tumor cells are characteristic of high proliferation, fluorescent staining of the proliferation marker Ki‐67 would provide information about the location of the malignant tumor cells in the tissue.^[^ ^53^ ^]^ Ki‐67 immunostaining showed strong fluorescence around the microvessels in the lung, liver and spleen in the saline group (Figure 6c), indicating the migration of the tumor cells from the subcutaneous carcinoma to different organs through the blood vessels. Encouragingly, the mice administrated with ICG‐PtMGs@HGd and further received the laser irradiation exhibited no metastatic sites in all the organs based on the both H&E and Ki‐67 immunostaining data (Figure 6c), indicating inhibited metastasis by ICG‐PtMGs@HGd.

### 2.9 Tumor Gene Expression Profile in Response to the Synergistic Phototherapy

**Figure 7:**
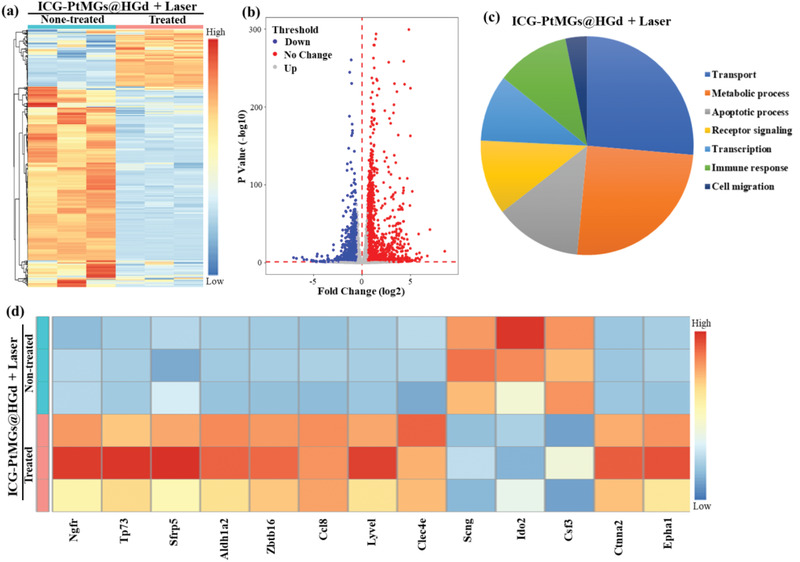
Altered tumor genetic profile in response to the synergistic phototherapy of ICG‐PtMGs@HGd nanoparticles under laser irradiation. a) Heatmap showing the hierarchical clustering of the differentially expressed genes (DEGs) in the mice treated with ICG‐PtMGs@HGd under laser irradiation (808 nm laser, 1.5 W cm^−2^, 5 min) compared with the nontreated (control) group. b) Volcano plots showing the up‐ and down‐regulated DEGs in the ICG‐PtMGs@HGd + Laser treated group compared to the control group. c) Pathway analysis of significantly altered genes after ICG‐PtMGs@HGd + Laser treatment. d) Selection of the potential genes in regulation of 4T1 tumor after the synergistic phototherapy by ICG‐PtMGs@HGd nanoparticles.

We further investigated the effects of synergistic phototherapy at the genetic level. RNA‐sequencing was performed to evaluate the gene expression profiles of the ICG‐PtMGs@HGd + laser and saline treated groups.^[^ ^54^ ^]^ To confirm the differentially expressed genes (DEGs), a row‐normalized dataset was generated with the standard of log_2_ fold‐change cutoff ≥1 and *p*‐value ≤0.05 according to the definition, and a hierarchical clustering of genes expressed after the combined phototherapy was calculated. A large number of DEGs were found in the ICG‐PtMGs@HGd + laser group compared to the control group (**Figure** **7**a), The discrepant clusters between the two groups suggested the effectiveness of the synergistic phototherapy. To identify the differential expression genes for the phototherapies, the volcano plots were developed, where the down and upregulated genes were exhibited with blue and red (left and right sides), among which 795 genes were upregulated while 935 genes were downregulated respectively (Figure 7b). We further performed gene ontology (GO) enrichment analysis to evaluate the possible biologic functions of the DEGs after ICG‐PtMGs@HGd + laser treatment, and found that the significantly enriched terms were mainly involved intracellular transport, metabolic process, apoptotic process, immune response, receptor signaling and transcription (Figure 7c), suggesting the effects of ICG‐PtMGs@HGd + laser treatment in these biological processes.

To identify interrelated altered genes related to the 4T1 breast cancer in response to the synergistic phototherapies, the correlated genes were analyzed from the candidates cancer gene database.^[^ ^55^ ^]^ Apoptotic‐related genes (i.e., nerve growth factor receipt (Ngfr), homo sapiens tumor protein p73 (TP73) and secreted frizzled‐related protein 5, (Sfrp5)) and the genes that negatively regulate cell proliferation (i.e., acetaldehyde dehydrogenase 2 (Aldhla2), zinc finger and BTB domain containing 16 (Zbtb16)) were obviously upregulated (Figure 7d), suggesting that ICG‐PtMGs@HGd + laser treatment induced the apoptosis of tumor cells and suppressed tumor growth, as was observed in cellular and in vivo experiments (Figures 5 and 6). The genes associated with intracellular transport such as membrane trafficking (i.e., *α*2 catenin (cadherin‐associated protein), Ctnna2) that was the structural constituent of cytoskeleton and cadherin binding and Epha1 (recombinant human ephrin A receptor 1) that was a protein‐tyrosine kinase) were also found to be upregulated (Figure 7d), indicating the phototherapy can induce the intracellular transport.^[^ ^54^ ^]^ Six immune‐related DEGs were also identified, among which three genes (chemokine (C–C motif) ligand 8 (Ccl8), lymphatic vessel endothelial hyaluronan receptor‐1 (Lyve1) and C‐type lectin domain family 4 member E (Clec4e)) that play antitumor immunogenic roles^[^ ^56^ ^]^ were upregulated and three gene (single copy nuclear gene (Scng), indoleamine 2,3‐dioxygenase 2 (Ido2), and colony stimulating factor 3 (Csf3)) that are related to cell‐mediated immunosuppressive responses^[^ ^57^ ^]^ were downregulated (Figure 7d), suggesting that the ICG‐PtMGs@HGd nanoparticle‐mediated synergistic phototherapy might cause antitumor immune response. Particularly, two genes (CCL8 and Clec4e) are involved mainly in the attraction and adhesion of immunocyte.^[^ ^56^, ^57^ ^]^ LYVE‐1, expressed in lymphatic endothelium, blood sinus endothelium, and certain macrophage lineages, are likely involved in lymph transportation.^[^ ^58^ ^]^ Three distinct downregulated genes (i.e., Scng, Ido2, Csf3) were mainly related to the immunosuppressive response for activation and proliferation of the main effector cells (i.e., dendritic cell, T cell) in cell‐mediated immunity. Overall, the six changed immune‐related DEGs showed that the synergistic phototherapy indeed gave rise to the immunological responses, which were also reported in the other PDT/PTT synergistic phototherapies.^[^ ^59^ ^]^ PDT has been demonstrated to induce immunogenic cell death (ICD) and activate an adaptive immune response against tumor‐associated antigens.^[^ ^60^ ^]^ As PDT is a typical oxygen‐consuming process whose effect can be severely limited by tumor hypoxia, thus the oxygen supplied nanoparticles can elicited ICD upon abundant ROS in PDT. Similarly, PTT has been shown to trigger the thermal ablation‐induced tumor ICD and cause the release of tumor antigens and endogenous adjuvants (e.g., heat shock proteins and damaged‐associated molecular patterns) to stimulate the immune responses.^[^ ^54^, ^61^ ^]^ Our ICG‐PtMGs@HGd nanoparticles provided an O_2_ self‐enriched nanoplatform for enhanced PDT/PTT, and might consequently enhance the synergistic phototherapy‐induced antitumor immune responses as the ICD caused by the phototherapy may trigger the maturation of dendritic cells and subsequently activated specific effector cells (e.g., CD8+ T cells, CD4+ T cells, macrophage polarization) to further inhibit the tumor growth and metastasis.^[^ ^62^ ^]^ However, more efforts would be put on the functional studies to further elucidate the synergistic phototherapy‐mediated immune responses.

### 2.10 Long‐Term Biotoxicity

**Figure 8:**
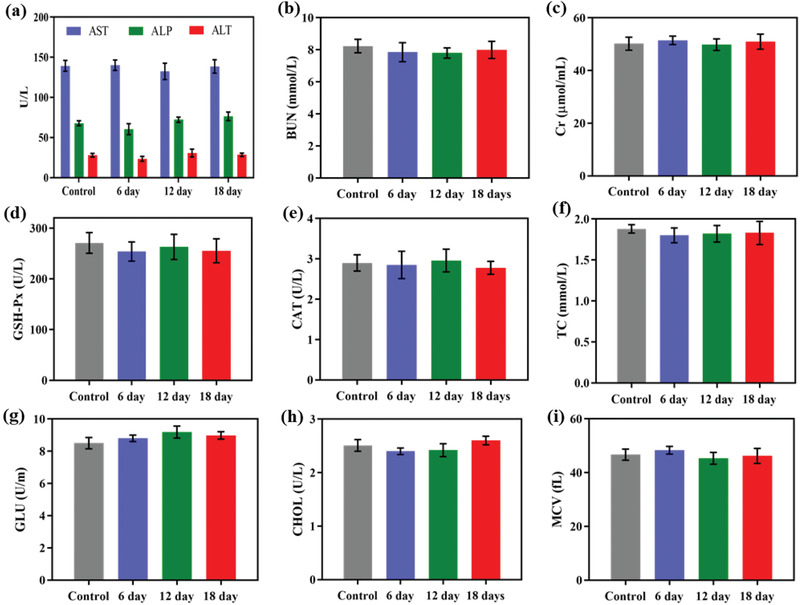
Long‐term toxicity of ICG‐PtMGs@HGd. Hematology and blood biochemical of a) AST, ALP, and ALT, and b–i) BUN, Cr, GSH‐PX, CAT, TC, GLU, CHOL, and MCV from the mice treated with ICG‐PtMGs@HGd nanoparticles at 6, 12, and 18 d. Data are presented as mean ± SD.

The routine in vivo blood biochemical analysis was comprehensively performed to evaluate the long‐term biotoxicity and biosafety of ICG‐PtMGs@HGd. The blood from the healthy mice was collected 6, 12, or 18 d after the intravenous injection of ICG‐PtMGs@HGd. Both the liver function index of ALT, ALP, and AST (**Figure** **8**a) and the kidney function index of BUN, Cr, CAT and GSH‐PX (Figure 8b–e) was found to be stable and remained within the reference range after the injection of ICG‐PtMGs@HGd, implying the compatible hepatic and kidney property of ICG‐PtMGs@HGd. Other parameters for the routine blood analysis such as (GLU, CHOL, MCV, and TC also fell within the normal ranges (Figure 8f–i), suggesting negligible in vivo blood toxicity of ICG‐PtMGs@HGd at proper treated dose.^[^ ^63^ ^]^ These results, together with the H&E staining of the main organs after the treatment with ICG‐PtMGs@HGd, that showed on noticeable organ impairment nor obvious inflammation or necrosis^[^ ^64^ ^]^ (Figure 6c) demonstrate the low systemic systematic toxicity and the superior biosafety of ICG‐PtMGs@HGd for the in vivo application.

## 3 Conclusion

We have successfully designed and synthesized ICG‐PtMGs@HGd nanoparticles to serve as a versatile nanotheranostics with a TME‐modulating effect for multimodal imaging‐guided synergistic enhanced PDT and PTT effect. The obtained ICG‐PtMGs@HGd nanoplatforms could continuously consume endogenous H_2_O_2_ inside the TME of the solid tumor by the contained Pt nanozyme, thereby elevating the concentration of oxygen in situ, which significantly alleviated tumor hypoxia and further enhanced PDT effect. Moreover, the nanoplatform exhibited a considerable PTT effect with high photothermal conversion efficiency due to the novel octahedral gold nanoshells. Corporation of the HGd hybrids provides the nanocomposites with high biocompatibility and excellent MRI ability. Furthermore, in vitro and in vivo assays revealed that the nanoplatform showed high biosafety as well as the desirable therapeutic effect in the oncolysis and metastasis inhibition of breast cancer. Therefore, we believe that this biomimetic multifunctional nanoplatform that integrates the synergistic treatments, imaging, and continuous modulation of hypoxia TME not only remarkably promotes more precise and effective cancer therapy, but also opens an avenue for the application of metal materials‐based nanozyme in the biomedical field.

## 4 Experimental Section

##### Materials

Zirconium chloride (ZrCl_4_) was purchased from Innochem Co., Ltd. 2‐aminoterephthalic acid (NH_2_‐BDC) and 11‐mercaptoundecanoic acid (MUA) were brought from Aladdin. Dimethyl formamide (DMF) and acetic acid were obtained from Beijing Chemical Works. Chloroplatinic acid (H_2_PtCl_6_·6H_2_O), chloroauric acid (HAuCl_4_), N‐(3‐(dimethylamino)‐propyl)‐3‐ethylcarbodiimide hydrochloride (EDC), indocyanine green (ICG), N‐hydroxysuccinimide (NHS), HSA, and Gd(NO_3_)_3_·6H_2_O were all obtained from J&K Scientific Co., Ltd. Potassium carbonate was obtained from VETEC (Sigma‐Aldrich). Formaldehyde was brought from Xilong Scientific Co., Ltd. 1,3‐Diphenylisobenzofuran (DPBF) and Ti(SO_4_)_2_ were obtained from Energy Chemical (Shanghai). PVP (*M*_w_ = 58 000), PVP (*M*_w_ = 40 000), 4′, 6‐diamidino‐2‐phenylindoles (DAPI), MTS cell proliferation colorimetric assay kit, calcein‐AM/PI double stained kit, annexin V‐FITC/PI apoptosis detection kit and DCFH‐DA were all obtained from Solarbio. Penicillin/streptomycin and Lyso‐Tracker‐Green DND‐26 were bought from Invitrogen (USA). Fetal bovine serum (FBS) and RPMI Media 1640 were purchased from Gibco Co., Ltd. HIF‐1*α* (Alexa Fluor 488 Conjugate) and phalloidin (Rhodamine Conjujugate) were bought from Abcam.

##### Instrument and Characteristics

Topography and elemental mapping images were recorded with Ht‐7700 transmission electron microscopy (Hitachi, Japan) and high resolution TEM (HRTEM) (Tecnai G2 F20, U.S.), respectively. UV–vis spectra were captured by Lambda UV–vis spectrophotometer (Perkin Elmer, US). Dynamic light scattering (DLS) for particle size and zeta potential were determined by Zetasizer Nano ZS (Malvern, UK). The XRD patterns were tested on a TZY‐XRD (Rigaku, Japan). The experiment of Barrett‐Joyner‐Halenda model from adsorption branch of isotherms was performed with automatic surface area and porosity analyzer (Micromeritics Instrument, US). The circular dichroism (CD) spectra were measured with circular dichroism chiroptical spectrometer spectropolarimeter system (Jasco J‐1500, Japan). Oxygen (O_2_) generation was determined with portable dissolved oxygen meter (Rex, JPB‐607A, China). The concentration of Au was determined by inductively coupled plasma mass spectroscopy (GGL‐ICP‐MS) (PerkinElmer, US). The laser of 808 nm was administrated by power‐tunable infrared laser (Laserwave, China). Thermal images were captured by using a DT‐980 infrared camera (Huashengchang Co., Ltd., China) and analyzed by infrared image software.

##### Preparation of PtMGs@HGd

PtMGs and MGs were fabricated first. Typically, H_2_PtCl_6_⋅6H_2_O (0.196 mmol) and 444 mg of poly(vinylpyrrolidone) (PVP, *M*_w_ = 58 000) were dissolved in 50 mL of ethylene glycol in a two‐neck round‐bottomed flask. The solution was reacted in an oil bath at 180 °C for 10 min, and the obtained PVP‐protected Pt nanoparticles were then precipitated in acetone after cooling down. Subsequently, the Pt nanoparticles were centrifugated (10 000 rpm, 10 min), cleaned with acetone and hexane to remove excess free PVP, and redispersed in DMF (20 mL) (with Pt nanoparticles concentration of about 1 mg mL^−1^) for further use.

The Pt‐decorated MOF (Pt‐MOFs, PtMs) nanoparticles were prepared based on the previous method with minor modification.^[^ ^26^ ^]^ Briefly, ZrCl_4_ (8.75 × 10^−3^ m) and NH_2_–BDC (8.01 × 10^−3^ m) were dissolved in DMF (5 mL) respectively and were subsequently mixed in the reactor followed by adding acetic acid (1.2 mL) and above Pt nanoparticles (2 mL). The whole mixture was sealed into the vacuum drying oven to crystallize at 120 °C for 12 h. After that, the obtained product was collected, washed by centrifugation with DMF for three times, then transferred into ethanol at 37 °C for 12 h and last dispersed in 5 mL of deionized water for following use. For preparation of MOFs without Pt decoration, we further added the same amount of ZrCl_4_, NH_2_–BDC and acetic acid (except for Pt nanoparticles), and all the other steps are the same.

The gold growth solution was first synthesized as follows: K_2_CO_3_ (50 mg) and HAuCl_4_ (50 × 10^−3^ m, 1.5 mL) were mixed in deionized water (100 mL) under stirring at 900 rpm for 10 min in the dark. Next, 1 mL of obtained PtMs solution was added into the gold growth solution followed by adding 10 mL of 1 mg mL^−1^ PVP (*M*_w_ = 40 000) and formaldehyde (2 mL). The mixture was then reacted for 2.5 min under stirring of 450 rpm.^[^ ^21^ ^]^ Last, the obtained PtMGs nanoparticles were collected and subsequently purified with water by centrifugation (10 000 rpm, 10 min) for several times. The MGs nanoparticles were prepared using MOFs as template, and all the other steps are the same.

The PtMGs‐COOH nanoparticles were then constructed. The PtMGs were scattered ethanol solution with ultrasound. After that, 11‐mercaptoundecanoic acid (MUA) with 10 times’ weight of PtMGs, was added for reaction of 12 h to get the PtMGs‐COOH nanoparticles. Last, the obtained nanoparticles were collected and purified with ethanol by centrifugation (10 000 rpm, 10 min). After that, the HGd hybrids was synthesized according to the reported method.^[^ ^65^ ^]^ Specifically, HSA (0.25 g) was dissolved in deionized water (9 mL) at 37 °C followed by adding of Gd(NO_3_)_3_ (50 × 10^−3^ m, 1 mL) aqueous solution for 5 min's reaction. Subsequently, NaOH solution (2 m, 1 mL) was rapidly introduced and stirred at 37 °C overnight. Last, HGd mixture was purified by dialysis method for 24 h.

Finally, PtMGs@HGd nanoparticles were prepared. Specifically, EDC (3.2 mg) and NHS (2.4 mg) were injected into PtMGs‐COOH solution (0.1 mg mL^−1^, 20 mL) for activating the carboxyl group. About 2 h later, the redundant compounds were discarded by centrifugation, and HGd hybrids (0.35 mL) was introduced for another 24 hours’ reaction. The obtained PtMGs@HGd were collected and further purified by centrifugation.

##### Preparation of ICG‐PtMGs@HGd

PtMGs@HGd (0.4 mg) were separately added into 1 mL ICG solution (50, 100, 200, 300, 400, and 500 µg mL^−1^, respectively) and then incubated for 24 h at room temperature. After that, the complex was washed with centrifugation for several times to remove unloaded ICG, then the supernatant and all the washing solution were collected to accept the absorbance measure at 780 nm for evaluating the loading capacity of ICG based on the following formula:

(1) $$\mathrm{Loading}\;\mathrm{capacity}=\left(M_{\mathrm{ICG}-}M_{\mathrm{uICG}}\right)/M_{\mathrm{ICG}-\mathrm{PtMGs}@\mathrm{HGd}}\times 100\%$$

where *M*_ICG_, *M*_uICG,_ and M_ICG‐PtMGs@HGd_ were the total mass of ICG, unloaded ICG and ICG‐PtMGs@HGd, respectively.

##### Release Behaviors of ICG Molecule

The drug leakage of ICG were detected based on the dialysis method at 37 °C in PBS buffers with different pH values (pH 7.4, 6.5, and 5.0). ICG‐PtMGs@HGd (8 mL) was put in a dialysis bag and then immersed into 60 mL PBS receptor fluid. For each predesigned time interval, 2 mL of the PBS solution was taken out and tested by UV–vis spectrum at the wavelength of 780 nm to quantify the amount of released ICG. After that, 2 mL of new PBS solution was further introduced to maintain consistence. All the release studies were performed in a dark environment.

##### Catalase Activity Detection of ICG‐PtMGs@HGd

The H_2_O_2_ consumptions were first investigated. The titanium sulfate (Ti(SO_4_)_2_) were used as an H_2_O_2_ indicator, whose color changes from colorless to yellow when specifically react with H_2_O_2_.^[^ ^33^ ^]^ The catalytic effect of ICG‐PtMGs@HGd was evaluated by mixing H_2_O_2_ (1 × 10^−3^ m) and ICG‐PtMGs@HGd (80 µg mL^−1^) in PBS (solution A) shaken at 37 °C. Ti(SO_4_)_2_ (50 mg) was dissolved into of H_2_SO_4_ (8.33 mL) and further diluted with deionized water to 50 mL volume (solution B). For every 30 min, 1 mL of solution A was centrifuged, then 0.35 mL of the supernatant was mix with 2.65 mL of solution B and its UV–vis absorbance at 405 nm was measured. The PBS and ICG‐MGs@HGd groups were also performed based on the former methods as control. Continuous catalytic behavior was tested by introducing the H_2_O_2_ to the ICG‐PtMGs@HGd for several repeated times, and the remaining concentration of H_2_O_2_ was detected with the same method.

To investigate the capability of oxygen generation with the intracellular H_2_O_2_, ICG‐PtMGs@HGd was ultrasonically scattered in water with 1 × 10^−3^ m H_2_O_2_, then the oxygen content was measured by a portable dissolved oxygen meter over time. In addition, the successive O_2_ production by introduce of H_2_O_2_ (1 × 10^−3^ m) into ICG‐PtMGs@HGd suspension every 30 min was also measured over time.

##### Measurement of Photothermal Performance

To explore the photothermal performance, PtMGs@HGd solution (0.5 mL) of various concentrations (0, 20, 40, 80, 160, and 320 µg mL^−1^, respectively) and 0.5 mL different formulations of PBS, HGd, PtMGs@HGd, ICG, and ICG‐PtMGs@HGd (ICG: 10 µg mL^−1^, PtMGs@HGd: 80 µg mL^−1^) were put into the eppendorf tubes, then exposed to laser irradiation (808 nm, 1.5 W cm^−2^, 5 min). The real time thermal images and the corresponding temperature quantitative value were determined by an infrared thermal camera. The photothermal stability of ICG‐PtMGs@HGd was performed with cyclical laser irradiation (808 nm, 1.5 W cm^−2^) for 420 s, then naturally cooled without irradiation for 600 s. This cycle was then repeated for four times. The photothermal conversion efficiency (*η*) was calculated based on the reported method.^[^ ^4c^ ^]^ Briefly, ICG‐PtMGs@HGd (ICG: 10 µg mL^−1^, PtMGs@HGd: 80 µg mL^−1^) was irradiated with a NIR laser (808 nm, 1.5 W cm^−2^) until the formulation reached a stable value, then the nanoparticle was naturally cooled to room temperature. The temperature changes during the whole process were recorded and the *η* value was calculated (Supporting information). To test whether photothermal effect can enhance the Pt catalyzed O_2_ generation, the oxygen generation of the PtMGs@HGd nanoparticles were measured with or without laser irradiation (808 nm, 1.5 W cm^−2^) using the dissolved oxygen meter under the introduction of H_2_O_2_.

##### Extracellular O_2_ Generation‐Enhanced PDT Effect

1,3‐Diphenylisobenzofuran (DPBF) was chosen to test the production of singlet oxygen by ICG‐PtMGs@HGd in the presence of H_2_O_2_.^[^ ^34^ ^]^ Briefly, 20 µL of DPBF (5 mmol L^−1^, acetonitrile) was mixed with 2 mL different formulations (PBS, PtMGs@HGd and ICG‐PtMGs@HGd (normoxia conditions); ICG‐PtMGs@HGd, ICG‐PtMGs@HGd + H_2_O_2_ and ICG‐PtMGs@HGd + H_2_O_2_ (hypoxia conditions) with the same amount of ICG (4 µg mL^−1^) and PtMGs@HGd (32 µg mL^−1^). The solutions were further exposed to NIR laser for 10 min, then ultraviolet absorption intensity of DPBF at 410 nm was recorded. Herein, hypoxic condition was achieved by blowing 1% oxygen into the solution for 30 min before the test.

##### Cell Culture and Cellular Uptake

4T1 cells were purchased from National Infrastructure of Cell Resource (Beijing) and cultured in RPMI‐1640 culture medium with 10% FBS and 1% penicillin‐streptomycin at 37 °C in a humidified incubator containing 5% CO_2_.

For cellular uptake studies, 4T1 tumor cells were planted into a CLSM dishes (4 × 10^5^ cells per well), then ICG and ICG‐PtMGs@HGd (with the same ICG concentration of 20 µg mL^−1^) were added for incubation of 1 and 6 h, respectively. The 4T1 cells were washed with PBS, sequentially stained with Lyso‐Tracker‐Green DND‐26 and DAPI, and further captured with confocal microscopy (Zeiss 710, Germany). The Pearson's correlation coefficient was obtained based on the Image J software.

##### Cytotoxicity Assays In Vitro

MTS experiment was first applied to test the cytotoxicity of the obtained ICG‐PtMGs@HGd formulations. 4T1 were preseeded into a 96‐well plate at the density of 5 × 10^4^ per well for overnight and further incubated with different concentrations of ICG‐PtMGs@HGd (0, 20, 40, 80, 160 and 320 µg mL^−1^ of PtMGs@HGd and 0, 2.5, 5, 10, 20, and 40 µg mL^−1^ of ICG) for 24 h. Then the culture medium was eliminated and 20 µL of MTS solution was introduced into each well and incubated for another 2 h. Finally, the absorbance at 490 nm of was measured using a microplate reader (Infinite M200, Switzerland). For exploring the cytotoxicity of the enhanced PDT/PTT effect in the normoxic and hypoxic tumor microenvironment, 4T1 cells were added with different formulations of PBS, PtMG@HGd, ICG, ICG‐PtMG@HGd, and ICG‐PtMG@HGd + H_2_O_2_ (ICG: 10 µg mL^−1^, PtMGs@HGd: 80 µg mL^−1^) for 6 h under normoxic and hypoxia condition (21% and 1% oxygen concentration, respectively).^[^ ^66^ ^]^ Next the cells of different groups were irradiated with an NIR laser (808 nm, 1.5 W cm^−2^, 5 min), then incubated for another 18 h and determined by MTS assay. The PBS group without laser irradiation was employed as control.

To observe the therapeutic effect vividly, the synergistic PDT/PTT effects of ICG‐PtMGs@HGd in hypoxic tumor microenvironment were further verified by AM/PI cell costaining assays. Specifically, 4T1 cells were preseeded into confocal dishes (1 × 10^6^ cells per well), then added with different preparations of PBS, PtMG@HGd, free ICG, ICG‐PtMG@HGd, and ICG‐PtMG@HGd + H_2_O_2_ (ICG:10 µg mL^−1^, ICG‐PtMGs@HGd: 80 µg mL^−1^) for 6 h under normoxic and hypoxia condition (21% and 1% oxygen concentration, respectively) before 5 min's laser irradiation. Subsequently, the tumor cells were washed with PBS, incubated with AM/PI kit and then observed by confocal fluorescence microscopy (Zeiss 710, Germany). The PBS group without laser irradiation was used as control.

To determine the apoptotic and necrotic cells, the 4T1 cells were preseeded into a six‐well plate for 24 h at a density of 1 × 10^6^ cells per well. Then the cells were incubated with different formulations of PBS, PtMG@HGd, free ICG, ICG‐PtMG@HGd, and ICG‐PtMG@HGd + H_2_O_2_ (ICG: 10 µg mL^−1^, ICG‐PtMGs@HGd: 80 µg mL^−1^) for overnight under hypoxia conditions (1% oxygen concentration) before NIR laser. Afterwards, 4T1 cells were collected, stained with annexin V/PI kit, and then detected by flow cytometer (BD Accuri C6, US). The PBS group without laser irradiation was used as control.

The inhibition effect of tumor metastasis was tested by cell migration assay according to a transwell model. Briefly, 4T1 cells were fed with PBS, free ICG, PtMGs@HGd, ICG‐PtMGs@HGd, and ICG‐PtMGs@HGd + H_2_O_2_ (ICG: 10 µg mL^−1^, ICG‐PtMGs@HGd: 80 µg mL^−1^) for 4 h, and irradiated with 2 min's NIR laser (808 nm, 1.5 W cm^−2^). Then the old medium was removed 24 later, and replaced with FBS‐free medium for another 6 h’ incubation. In addition, 4T1 cells were washed, digested, and put into the upper chambers of a transwell with FBS‐free medium (100 µL), then the culture medium containing FBS was added into the lower chambers. Last, 4T1 cells in the upper chambers were wiped off and washed after 24 h, then the membrane was stained with 0.1% crystal violet and observed by a microscope for migrated cells.

##### Detection of Intracellular H_2_O_2_ Catalytic Activity, O_2_, Cellular Hypoxia and ROS Generation

MAK164 (Sigma Aldrich), an intracellular H_2_O_2_ assay, was used to evaluate the intracellular H_2_O_2_ concentration.^[^ ^21^ ^]^ Typically, 4T1 cells were seeded on the chambered cover glass, and then incubated with PBS or ICG‐PtMGs@HGd (45 and 90 µg mL^−1^) under normal or hypoxic condition (21% and 1% oxygen concentration) for 24 h and washed with PBS for several times. After that 100 µL DMEM medium containing 100 × 10^−6^ m H_2_O_2_ was added and incubated for 1 h. In addition, the cell medium was discarded and washed followed by incubation with MAK164 working solution for another 1 h. After washing with PBS, the intracellular H_2_O_2_ concentration was evaluated by fluorescent microscope (ex/em = 490/520).

For detecting the intracellular O_2_ generation, 4T1 cells were seeded on the chambered cover glass. Then, the cells in each group were incubated with PBS or ICG‐PtMGs@HGd under normal or hypoxic condition (21% and 1% oxygen concentration) for 24 h. These cells were reacted with [Ru(dpp)_3_]Cl_2_ (10 µg mL^−1^) (Sigma‐Aldrich) for another 12 h followed by rinsing with PBS to remove free [Ru(dpp)_3_]Cl_2_ and the residual particles.^[^ ^35^ ^]^ The intracellular oxygen level was detected based on the fluorescence of [Ru(dpp)_3_]^2+^ (ex/em = 450/610 nm).

The cellular hypoxia condition under normoxic or hypoxic environments was evaluated following treated with PBS or ICG‐PtMGs@HGd (45 and 90 µg mL^−1^). 4T1 cells were fixed with paraformaldehyde, permeabilized with PBS containing 0.2% Triton X‐100 and then blocked with PBS containing 0.05% Tween‐20 buffer and 1% bovine serum albumin. After that, the tumor cells were incubated with anti‐HIF‐1*α* antibody (Abcam, ab179483) at 4 °C overnight, washed with PBS containing 0.2% Triton X‐100 and 2% Tween‐20 buffer for three times, and incubated with CoraLite488‐conjated AffiniPure Goat Anti‐Rabbit LgG (Proteintech, SA00013‐2) for 1 h at room temperature. Finally, the cells were further washed with PBS containing 0.2% Triton X‐100 and 2% Tween‐20 buffer for three times, incubated with rhodamine conjugate‐phalloidin (Abcam, ab235138) at room temperature for 1 h and then observed by CLSM.

DCFH‐DA was selected as a typical ROS indicator to detect intracellular ROS of ICG‐PtMGs@HGd in 4T1 cells.^[^ ^37^ ^]^ Briefly, 4T1 cells were planted into CLSM dishes (1 × 10^6^ cells per well), and further fed with different preparations (PBS, free ICG, ICG‐PtMGs@HGd and ICG‐PtMGs@HGd + H_2_O_2_, respectively) at 37 °C under the normoxia or hypoxia condition (21% and 1% oxygen concentration). After incubation for 4 h, the cells were washed with PBS, exposed to NIR laser (808 nm, 1.5 W cm^−2^) for 3 min, incubated with DCFH‐DA (10 × 10^−6^ m) for 30 min, then observed by CLSM.

##### Penetrability of ICG‐PtMGs@HGd

Multicellular cancer spheroids (MCSs) was constructed to perform an intermediary between the 2D monolayer cell model and the solid tumor model.^[^ ^67^ ^]^ Typically, 50 µL of 1% (w/v) agarose solution was introduced into a 96‐well plate and exposed to UV irradiation for 30 min. Then 1000 cells (200 µL) cell was added and further incubated for one week to form the 4T1 MCSs. To investigate the penetrability, the tumor spheroids were fed with free ICG (20 µg mL^−1^) or ICG‐PtMGs@HGd (ICG: 20 µg mL^−1^, ICG‐PtMGs@HGd: 160 µg mL^−1^). After incubation for 24 h, the spheroids were put into CLSM culture dishes, washed with PBS and observed with CLSM with Z‐stack scanning.

##### Animals and Tumor Models

The female nude mice and BALB/c mice (6–8 weeks old) were bought from Charles River (Beijing, China). *In vivo* tumor animal model was obtained by subcutaneously inoculating ∼2 × 10^6^ 4T1 cells into the right flank part. When the tumor volume reached about 150 mm^3^, the animal experiments could be carried out in accordance with the guidelines approved by the Ethics Committee of the National Center for Nanoscience and Technology of China.

##### Multimodal Imaging In Vitro and In Vivo

In order to investigate the fluorescence imaging, the tumor cells‐bearing mice were administrated with free ICG and ICG‐PtMGs@HGd (1.0 mg kg^−1^ of ICG) by intravenous injection. After that ICG fluorescence images were acquired at the predesigned time points by IVIS Spectrum imaging system (Perkin Elmer, USA). At the time point of 24 h, mice were sacrificed, then the major organs and tumors were collected for ex vivo fluorescence imaging and quantitative biodistribution analysis.

The multispectral optoacoustic intensity of ICG‐PtMGs@HGd in vitro was first measured at various ICG concentrations (0, 5, 10, 20, 40, and 80 µg mL^−1^) by the InVision 128 MSOT system (iThera Medical, Germany). In order to test the in vivo multispectral optoacoustic imaging, 200 µL of ICG‐PtMGs@HGd (1.0 mg kg^−1^ of ICG) was injected into the tumor‐bearing mice intravenously, then the mice were anesthetized and scanned at predesigned time points (0, 2, 6, 12, and 24 h) by MSOT system.

The hounsfield units (HU) signal of ICG‐PtMGs@HGd in vitro was first carried out at various Au content (0, 0.625, 1.25, 2.5, 5, 10 mg mL^−1^) with X‐ray CT (GMI, USA). To obtain the in vivo CT imaging, the ICG‐PtMGs@HGd (20 mg kg^−1^) was administrated into the tumor sites and the CT images were recorded after 12 h injection.

The in vitro T_1_‐weighted MR images of ICG‐PtMGs@HGd and Gd‐DTPA with the same amount of Gd were detected at various Gd concentrations (0 × 10^−6^, 25 × 10^−6^, 50 × 10^−6^, 100 × 10^−6^, 200 × 10^−6^, and 400 × 10^−6^ m, respectively) by 7.0 T small animal MRI instrument (BioSpec 70/20 USR, Breker, Germany). To test in vivo MR images, the tumor‐bearing mice were injected with ICG‐PtMGs@HGd (0.05 mmol Gd kg^−1^) by intravenous injection, then the MR images were acquired at predesigned time points (0, 2, 6, 12, and 24 h).

##### Biodistribution In Vivo

In order to investigate the biodistribution of ICG‐PtMGs@HGd, tumors and major organs (heart, liver, spleen, lung, and kidney) of ICG‐PtMGs@HGd (9 mg kg^−1^)‐injected mice were solubilized with aqua regia to get Au and Pt content by ICP‐MS test after 24 h injection. To test the blood circulation, the blood samples (200 µL) were collected at specific time points, weighted, and then solubilized with nitrohydrochloric acid. Last, the Au contents of the different samples were investigated by ICP tests to obtain the bloodstream pharmacokinetics profile.

##### Intratumoral Penetration, Hypoxia, and PTT Effect

To study the diffusion of ICG‐PtMGs@HGd under tumor microenvironment, the tumor bearing mice were treated with free ICG and ICG‐PtMGs@HGd (ICG, 1 mg kg^−1^) by intravenous injection. About 24 h later, mice were sacrificed, then tumors were excised, cut into proper slices, incubated with anti‐CD31 antibody (Abcam, ab28364) and DAPI, and further observed on a LSCM.

For investigating the intratumoral hypoxia, tumor bearing mice were intravenously injected with PBS, ICG‐MGs@HGd and ICG‐PtMGs@HGd, respectively. About 24 h later, the mice were sacrificed, then the tumors were excised, cut into slices, stained with anti‐HIF‐1 alpha (HIF‐1*α*) ((Abcam, ab1) and DAPI, and further observed under a LSCM.

In order to investigate in vivo photothermal effect, NIR laser (808 nm, 1.5 W cm^−2^) was applied at the tumor parts of the tumor‐bearing mice after intravenous injection of PBS, ICG, PtMGs@HGd, ICG‐MGs@HGd, and ICG‐PtMGs@HGd (1 mg kg^−1^ of ICG, 8 mg kg^−1^ of PtMGs@HGd) for 12 h. The thermographic images of the tumor sites were recorded by an infrared thermal camera to exhibit the temperature changes.

##### Therapeutic Efficacy and Metastasis of ICG‐PtMGs@HGd in Vivo

After the tumor volume reached ≈150 mm^3^, the mice were randomly separated into six groups: a) saline; b) saline + laser; c) PtMGs@HGd + Laser; d) Free ICG + laser; e) ICG‐MGs@HGd + Laser; f) ICG‐PtMGs@HGd + Laser. Each treatment with the same amount of ICG (1 mg kg^−1^) and Au (4 mg kg^−1^, derived from 8 mg kg^−1^ of PtMGs@HGd and MGs@HG, respectively) was injected from tail‐vein, and the photothermal therapy (808 nm, 1.5 W cm^−2^) was performed for 5 min after12 h injection. Tumor size and body weight of mice were measured every other day after irradiation. The tumor volume of the mice could be calculated according to the reported formula (volume = width^2^ × length/2).^[^ ^68^ ^]^ On the day of 18, mice of each group were sacrificed and the major organs as well as the tumors were harvested. The obtained tumors were weighted, imaged, and further processed forhematoxylin/eosin (H&E) staining and TUNEL staining. As a separate experiment, the survival rate of the mice which were treated under the same conditions was tested. To evaluate the inhibited effect of tumor metastasis, the obtained organs were disposed with H&E and ki67 staining to investigate the extent of tumor metastasis.

##### RNA Isolation and Sequencing

The tumors of saline and ICG‐PtMGs@HGd + Laser groups were stripped. The responding total RNA was extracted for the subsequent RNA sequencing (RNASeq) test. All the RNA‐Seq reads were mapped and the human genome sequence (Genome Reference Consortium GRCh37, hg19) were used as a reference. The differentially expressed genes (DEGs) were investigated by the softwares HTSeq and DEseq2. The transcript counts and the relative abundance of gene expression were calculated based on the software Cufflinks (version 2.1.1).

##### Long‐Term Toxicity Assessment In Vivo

To evaluate the long‐term toxicity of the obtained formulation, healthy Balb/c mice were intravenously administrated with saline and ICG‐PtMGs@HGd (ICG: 1 mg kg^−1^, PtMGs@HGd: 8 mg kg^−1^). Then 1 mL blood from each mouse was collected for the blood biochemistry analysis 6, 14, and 18 d postinjection. Liver function was tested by measuring serum levels of ALP (alkaline phosphatase), AST (aspartate transaminase), and ALT (glutamate transaminase). Kidney function was evaluated by determining BUN (blood urea nitrogen), Cr (creatinine), CAT (catalase), and GSH‐PX (glutathione peroxidase) content. Other parameters such as GLU (glucose), MCV (mean corpusular volume), TC (total cholesterol), and CHOL (cholesterol) were also tested to further testify the long‐term toxicity of ICG‐PtMGs@HGd.

##### Statistical Analysis

Quantitative data were exhibited as means ± standard deviation and performed by ANOVA. Differences among multiple groups were analyzed by one‐way ANOVA. Student‐ Newman‐Keuls test was utilized as post hoc test. *P* < 0.05 was identified to be statistically significant.

## Conflict of Interest

The authors declare no conflict of interest.

## Supporting information

Supporting Information
