# Supplementary material for: Persistent Regulation of Tumor Hypoxia Microenvironment via a Bioinspired Pt‐Based Oxygen Nanogenerator for Multimodal Imaging‐Guided Synergistic Phototherapy
Source: Adv Sci (Weinh). 2020 Jul 29;7(17):1903341. doi: 10.1002/advs.201903341 (PMC7507529; doi:10.1002/advs.201903341)
Supplement: Supplementary file 1 — Supporting Information [file ADVS-7-1903341-s001.pdf]

© 2020 Wiley-VCH GmbH

ADVANCED SCIENCE
Open Access

Supporting Information

for *Adv. Sci.*, DOI: 10.1002/advs.201903341

Persistent Regulation of Tumor Hypoxia Microenvironment via a Bioinspired Pt-Based Oxygen Nanogenerator for Multimodal Imaging-Guided Synergistic Phototherapy

Qing You, Kaiyue Zhang, Jingyi Liu, Changliang Liu, Huayi Wang, Mengting Wang, Siyuan Ye, Houqian Gao, Letian Lv, Chen Wang, Ling Zhu, and Yanlian Yang**

Supporting Information

Persistent Regulation of Tumor Hypoxia Microenvironment via the Bioinspired Pt-Based Oxygen Nanogenerator for the Multimodal Imaging-Guided Synergistic Phototherapy

Qing You,[†,‡] Kaiyue Zhang,[‡,§] Jingyi Liu,[†,‡] Changliang Liu,[†,‡] Huayi Wang,[†,⫽] Mengting Wang,[†,‡] Siyuan Ye,[†,⫽] Houqian Gao,[†,‡] Letian Lv,[†,‡] Chen Wang,[†,‡] Ling Zhu,[*,†] and Yanlian Yang[*,†,‡]

[†]CAS Key Laboratory of Standardization and Measurement for Nanotechnology, CAS Key Laboratory for Biomedical Effects of Nanomaterials and Nanosafety, CAS Center for Excellence in Nanoscience, National Center for Nanoscience and Technology, Beijing 100190, China

[‡]University of Chinese Academy of Sciences, Beijing 100049, P. R. China

[§]Sino-Danish College, University of Chinese Academy of Sciences, Beijing 100049, P.R. China

[⫽]Department of Chemistry, Tinghua University, Beijing 100084, P.R. China

Email: zhul@nanoctr.cn, yangyl@nanoctr.cn

Calculation of the photothermal conversion efficiency

The photothermal conversion efficiency of ICG-PtMGs@HGd nanoparticles was measured according to previous report.[1] ICG-PtMGs@HGd nanoparticles (ICG concentration of 10 μg/mL and PtMGs@HGd concentration of 80 μg/mL) underwent continuous irradiation of 808 nm laser (1.5 W/cm^2) until steady state temperature was reached. Then the laser was shut off, and the aqueous solution was naturally cooled to the environment temperature. The temperature change of the aqueous solution was recorded immediately (Figure S7a). The η value was calculated as follows:

$$\eta = \frac{hS(T_{max}-T_{Surr}) - Q_S}{I\,(1-10^{-A_{808}})} \times 100\% \qquad (1)$$

here h is the heat transfer coefficient, S is the surface area of the container, and the value of hS is obtained from the Equation (4) and Figure S7b. The maximum steady temperature (T_{max}) and environmental temperature (T_{Surr}) were 58.4°C and 22.0°C, respectively. The laser power for irradiation was 1.5 W/cm^2. The absorbance of the PUA nanoparticles at 808 nm A_{808} was 0.160. Q_S was heat dissipated from the light absorbed by the solvent and container. A dimensionless parameter θ was calculated as followed:

$$\theta = \frac{T - T_{Surr}}{T_{max}-T_{Surr}} \qquad (2)$$

A sample system time constant τ_s coul be calculated as Eq.3.

$$t = -\tau_s \ln(\theta) \qquad (3)$$

According to Fig.4b, τ_s was determined and calculated to be 168.3 s.

$$hs = \frac{m_D\text{-}C_D}{\tau_s} \times 1000 \qquad (4)$$

In addition, m_D was 0.2 g and C_D was 4.2 J/g·°C. Thus, according to Equation (4), hS was calculated to be 4.991 mW/°C.

Qs was heat dissipated from the light absorbed by the container itself, which was determined independently to be 3.528 mW using a container containing pure water. Thus, substituting according values of each parameters to Equation (1), the 808 nm laser photothermal conversion efficiency (η) of the ICG-PtMGs@HGd nanoparticles could be calculated to be 38.55%.

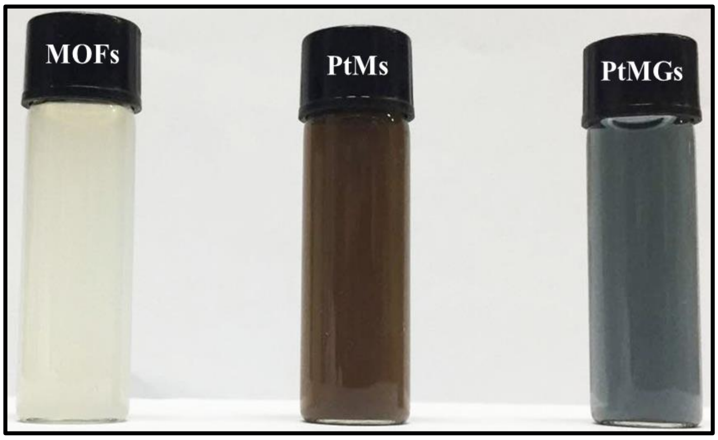

Figure S1. Photos of different nanocomposites (MOFs, PtMs and PtMGs, respectively) during the synthesis process of the PtMGs.

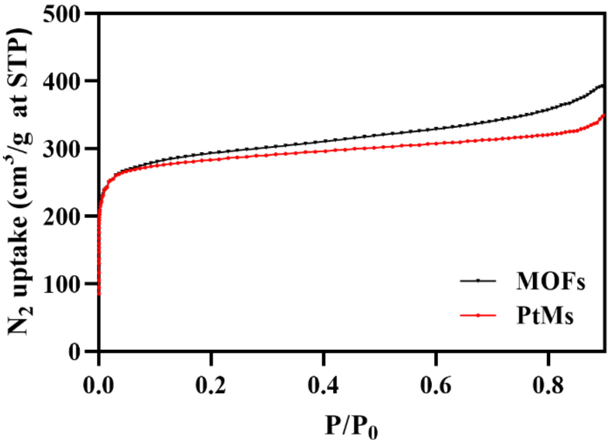

Figure S2. The nitrogen adsorption-desorption isotherm of different nanocomposites (MOFs and PtMs, respectively).

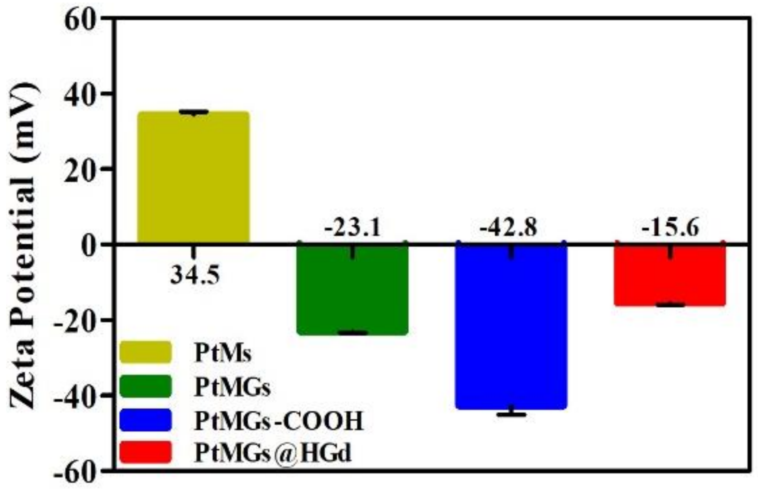

Figure S3. Zeta potential of different formulations (PtMs, PtMGs, PtMGs-COOH and PtMGs@HGd nanoparticles).

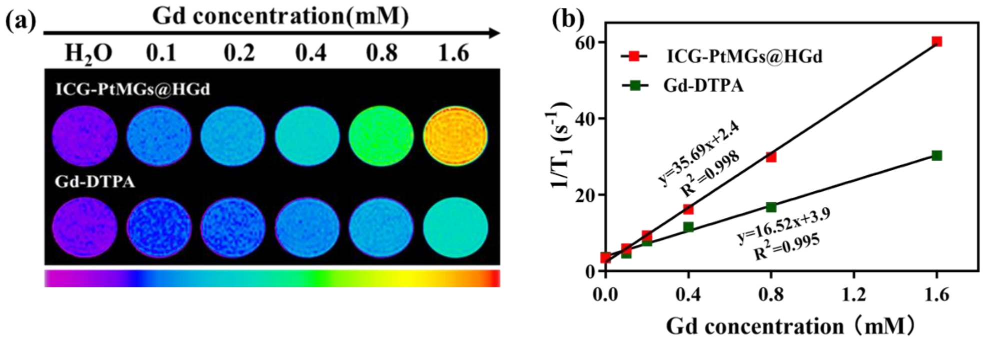

Figure S4. MR enhancement effects of ICG-PtMGs@HGd and Gd-DTPA. a) Color-coded (by signal intensity) T_1-weighted MR images of ICG-PtMGs@HGd and Gd-DTPA at varying Gd concentrations. b) The linear relationship between the T_1 relaxation rate ($1/T_1$) and Gd concentrations of ICG-PtMGs@HGd and Gd-DTPA solutions.

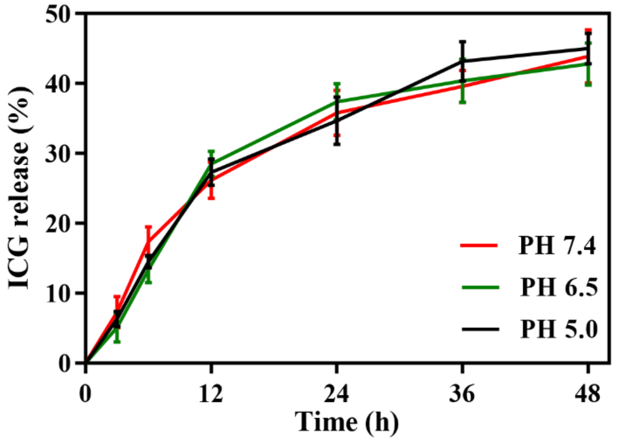

Figure S5. Release behaviors of ICG from ICG-PtMGs@HGd nanoparticles in PBS buffers with different pH values (pH 7.4, 6.5, and 5.0) at 37 °C.

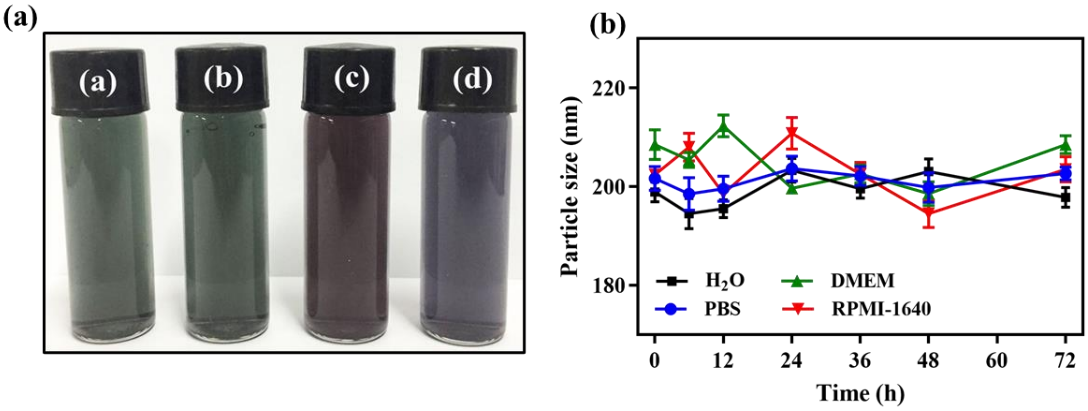

Figure S6. a) Photos of the ICG-PtMGs@HGd nanoparticles dispersed in different physiological solutions: H_2O (a), PBS (b), DMEM cell culture medium (c), and RPMI-1640 cell culture medium plus 10% fetal bovine serum (d) after 72 h. b) Size distribution changes of ICG-PtMGs@HGd nanoparticles in the different media (H_2O, PBS, DMEM cell culture medium, and RPMI-1640 cell culture medium plus 10% fetal bovine serum) at designed time points during 72 h measured by dynamic light scattering (DLS).

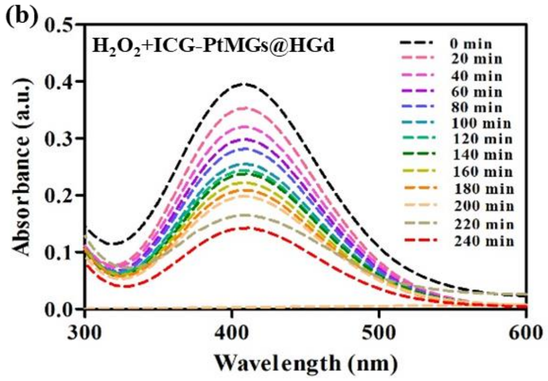

Figure S7. UV-vis absorption spectra of H_2O_2-Ti $(SO_4)_2$ solution treated with ICG-PtMGs@HGd nanoparticles at various time points.

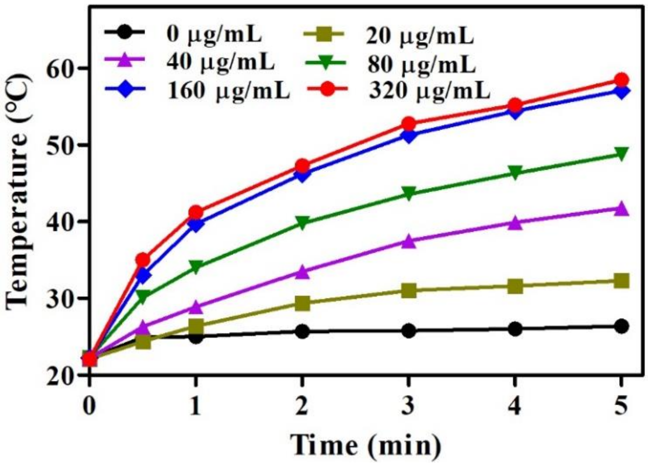

Figure S8. Laser-triggered temperature elevation of PtMGs@HGd nanoparticles at different concentrations over a period of 5 min laser irradiation (808 nm, 1.5 W/cm^2).

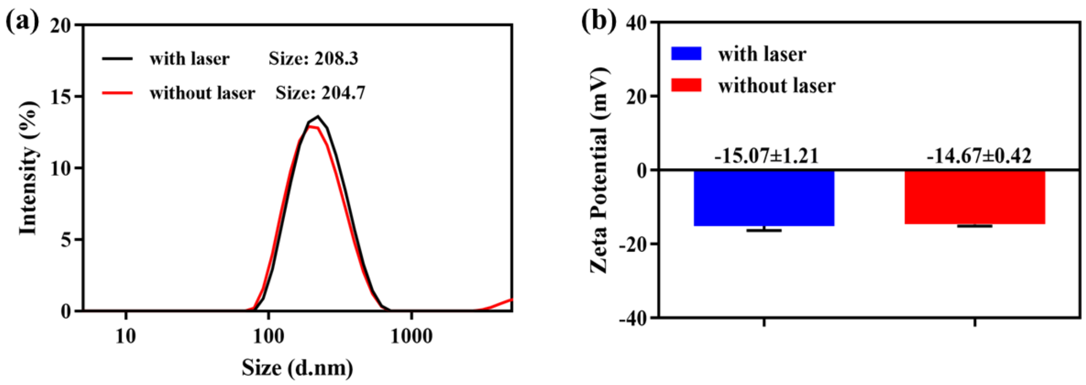

Figure S9. a) Size distribution and b) zeta potential of PtMGs@HGd nanoparticles with or without laser irradiation.

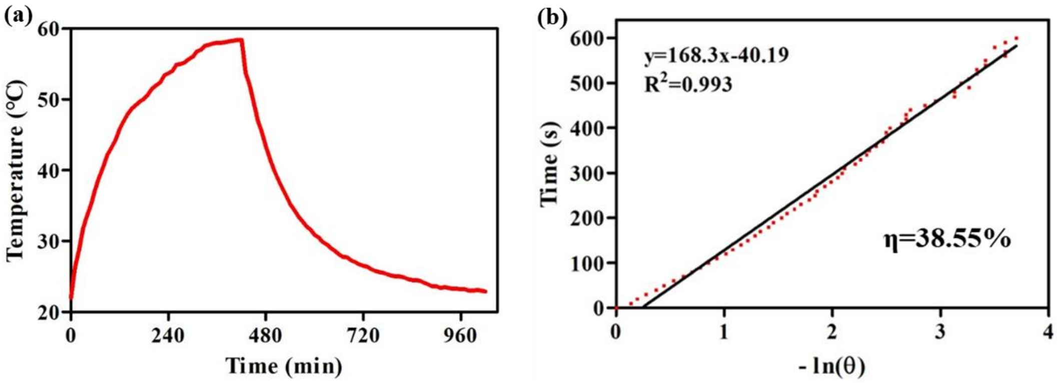

Figure S10. a) Photothermal effect of the aqueous solution of the ICG-PtMGs@HGd nanoparticles under laser irradiation (808 nm, 1.5 W/cm^2), where the laser was first irradiated for 420 s and then removed. b) Linear time data vs -ln(θ) obtained from the cooling period of Figure S7 a).

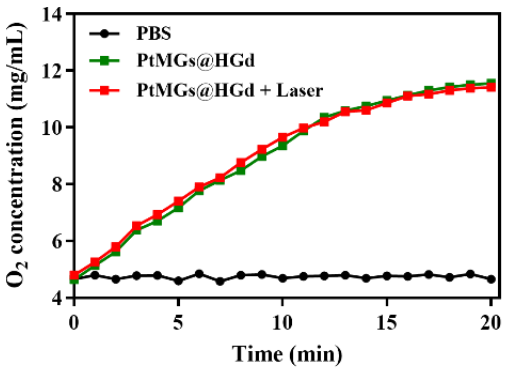

Figure S11. O_2 generation after treated with PBS, PtMGs@HGd and PtMGs@HGd + Laser at pre-designed time points. (Laser: 1.5 W/cm^2, 808 nm, 5 min)

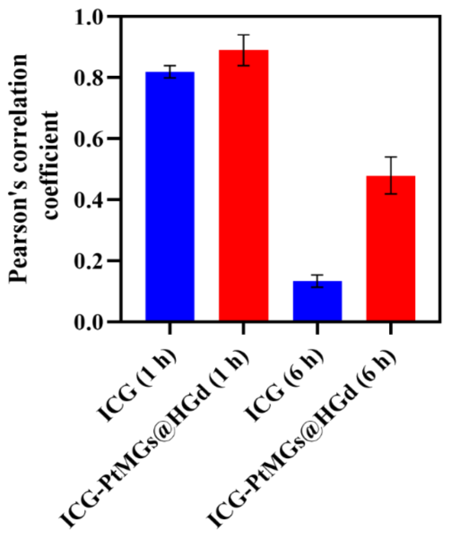

Figure S12. Pearson’s correlation coefficient showing the colocalization of LysoTracker Green and ICG-PtMGs@HGd in **Figure 3a** as calculated by Image J software.

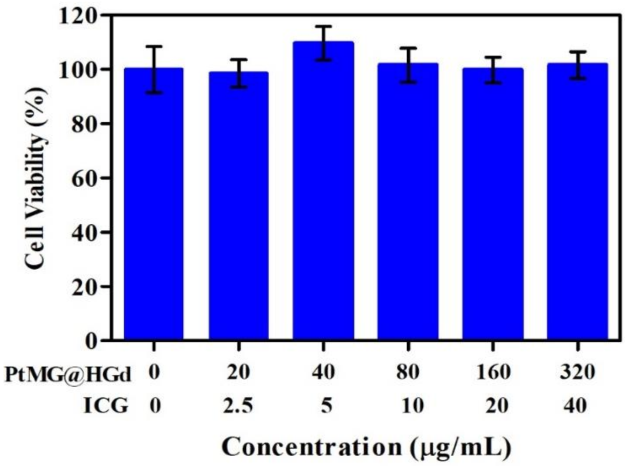

Figure S13. 4T1 cell viability after treatment with various concentrations of ICG-PtMGs@HGd nanoparticles as determined by MTS assay. Data are presented as means ± SD (n = 3).

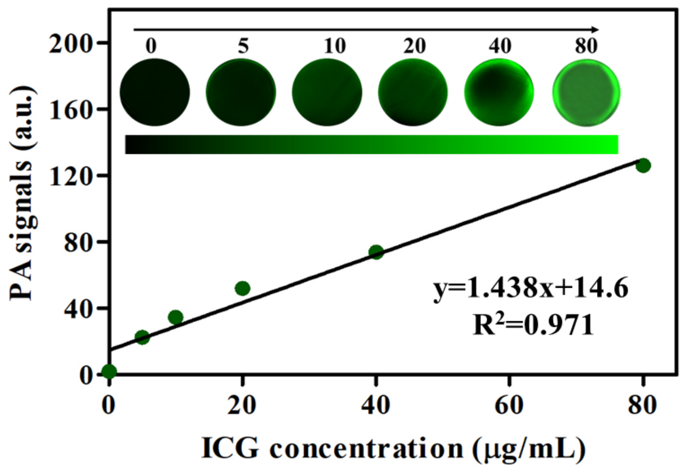

Figure S14. Photoacoustic intensity linearly fits to the concentration of ICG-PtMGs@HGd; inset: the corresponding MSOT images.

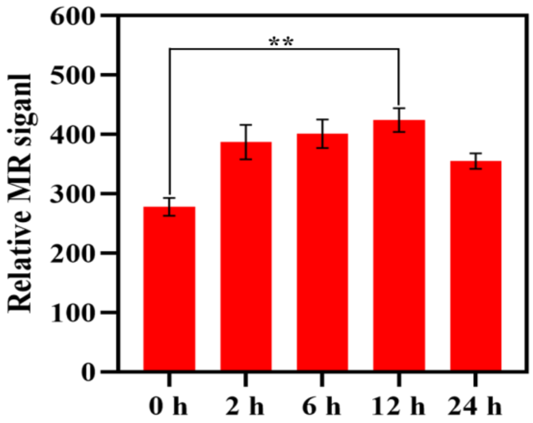

Figure S15. The normalized signal intensity of T_1-weighted MR signals from the tumor at different time post injection, $^{**}p < 0.01$ (Student's t-test).

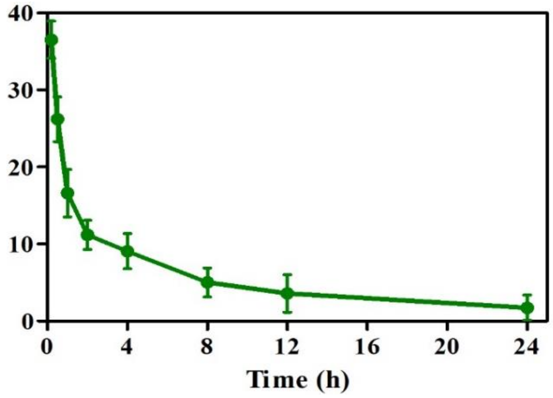

Figure S16. Pharmacokinetic profiles of ICG-PtMGs@HGd nanoparticles after intravenous injection based on Au concentrations.

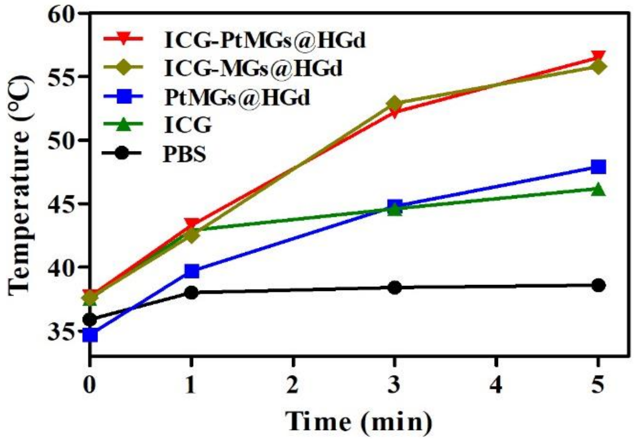

Figure S17. Tumor temperature rise curves of mice injected with different formulations (Saline, ICG, PtMGs@HGd, ICG-MGs@HGd and ICG-PtMGs@HGd, respectively) as a function of the irradiation time.

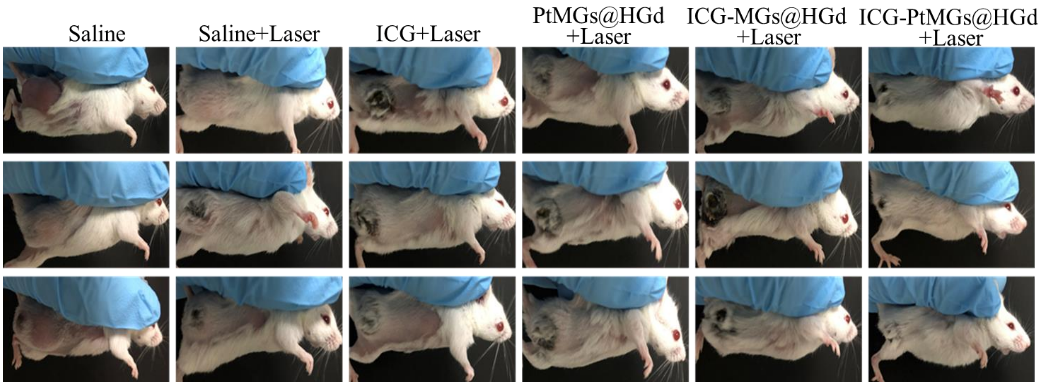

Figure S18. Representative photos of 4T1 tumor-bearing mice 18 days after various treatments.

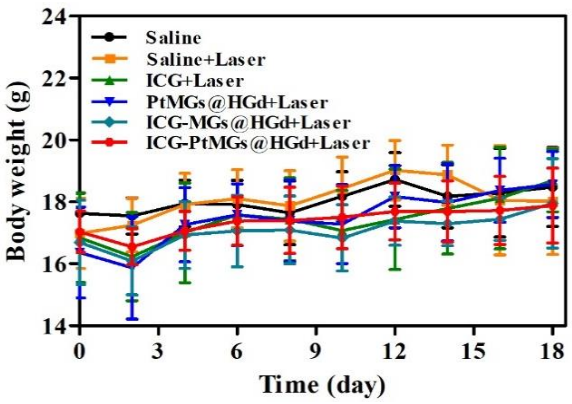

Figure S19. Body weight changes of 4T1 tumor-bearing mice as a function of days post treatments of various formulations.

Reference:

1. Q. You, Q. Sun, J. Wang, X. Tan, X. Pang, L. Liu, M. Yu, F. Tan, N. Li, *Nanoscale* **2017**, *9*, 3784.
